# Ovarian tumor cell-derived JAGGED2 promotes omental metastasis through stimulating the Notch signaling pathway in the mesothelial cells

**DOI:** 10.1038/s41419-024-06512-0

**Published:** 2024-04-04

**Authors:** Syed S. Islam, Falah H. Al-Mohanna, Iman M. Yousef, Ismail A. Al-Badawi, Abdelilah Aboussekhra

**Affiliations:** 1 Department of Molecular Oncology, King Faisal Specialist Hospital & Research Centre, Riyadh, Saudi Arabia; 2 School of Medicine, Al-Faisal University, Riyadh, Saudi Arabia; 3 Department of Comparative Medicine, King Faisal Specialist Hospital & Research Centre, Riyadh, Saudi Arabia; 4 Department of Obstetrics and Gynecology, King Faisal Specialist Hospital & Research Centre, Riyadh, Saudi Arabia

**Keywords:** Ovarian cancer, Ovarian cancer

## Abstract

The primary site of metastasis for epithelial ovarian cancer (EOC) is the peritoneum, and it occurs through a multistep process that begins with adhesive contacts between cancer cells and mesothelial cells. Despite evidence that Notch signaling has a role in ovarian cancer, it is unclear how exactly it contributes to ovarian cancer omental metastasis, as well as the cellular dynamics and intrinsic pathways that drive this tropism. Here we show that tumor cells produced the Notch ligand Jagged2 is a clinically and functionally critical mediator of ovarian cancer omental metastasis by activating the Notch signaling in single-layered omental mesothelial cells. In turn, Jagged2 promotes tumor growth and therapeutic resistance by stimulating IL-6 release from mesothelial cells. Additionally, Jagged2 is a potent downstream mediator of the omental metastasis cytokine TGF-β that is released during omental destruction. Importantly, therapeutic inhibition of Jagged2-mediated omental metastasis was significantly improved by directly disrupting the Notch pathway in omental mesothelial cells. These findings highlight the key role of Jagged2 to the functional interplay between the TGF-β and the Notch signaling pathways during the metastatic process of ovarian cancer cells to the omentum and identify the Notch signaling molecule as a precision therapeutic target for ovarian cancer metastasis.

## Background

Ovarian cancer (OvCa), the most lethal form of gynecological malignancy, is highly heterogeneous and complex with more than 70% of patients diagnosed with metastasis [1, 2]. By contrast to other solid tumors, high-grade serous ovarian cancer (HGSOC) rarely metastasizes hematogenously [1, 3]. Indeed, OvCa cell dissemination is a passive process, during which cancer cells detach from the primary tumor site, actively disseminate to the peritoneal fluid, and then anchor to the peritoneal surface omentum, the most typical and favored OvCa metastatic site [3–5]. The development of omental tumor growth depends on cross-communication between ovarian tumor cells and the omental mesothelial cells microenvironment. Upon contact, the tumor cells gain the ability to disrupt the omental homeostasis balance maintained by mesothelial cells and adjacent fibroblast cells. The tumor microenvironment (TME) in the omentum, mostly constituted of cancer-associated fibroblasts and adipocytes, supports the OvCa metastasis through cross-talk signaling [6, 7]. When OvCa cells adhere to and interact with mesothelial cells, several events take place simultaneously. First, by altering the expression of surface adhesion molecules and ligands, mesothelial cells are reprogrammed and stimulated to take on mesenchymal phenotypes, via a process called mesothelial-to-mesenchymal transition (MMT), and to promote a variety of tumorigenic activities, including invasion and migration [3, 8–10]. Previous studies have shown that several surface molecules, such as CD44 and α5β-integrin, promote the contact of ovarian cancer cells with mesothelial cells. Furthermore, secreted transforming growth factor-β (TGF-β) induces pro-tumorigenic alterations in mesothelial cells [8, 9, 11]. Despite these evidences, the molecular mechanisms governing OvCa cells’ preference for implantation into the omentum, and homeostasis, as well as their proactivity to favor disease progression, remain largely unknown.

The Notch signaling pathway controls several elements of cancer biology and cell fate determination. Studies have revealed that the Notch pathway is implicated in ovarian tumor development and adhesion [12] and is critical for both cancer cells and their TME. The Notch signaling pathway is largely activated by physical cell-cell contact between the signal-sending Notch ligand and the signal-receiving Notch receptor. The Notch ligands Jagged and Delta attach to Notch receptors to begin the Notch pathway’s signaling process. This releases the Notch receptors’ intracellular domain by a cascade of proteolytic cleavage, which is partly mediated by γ-secretase, and activates the HES and HEY family members of the Notch signaling pathway.

The epithelial-to-mesenchymal transition (EMT) process, angiogenesis, and the control of cancer stem cells (CSCs) are all significantly impacted by the Notch signaling pathway [13–15]. In ovarian cancer, the Notch ligand Jagged2 is associated with and linked to immune evasion in the microenvironment [16]. Despite these remarkable advances, the functional and regulatory involvement of the Notch signaling pathway in OvCa omental metastasis remains unknown. Furthermore, in order to develop effective therapies for the prevention of omental metastasis, a clear understanding of the complicated molecular and cellular network governing the interaction between the tumor and omental mesothelial cells in OvCa omental metastasis remains to be addressed. In this study, we have investigated the functional role of Notch signaling in the development of omental metastasis of ovarian cancer. These findings might pave the way for the development of novel anti-OvCa metastatic treatment agents that target the Notch signaling pathway to prevent and manage ovarian cancer metastasis.

## Materials and methods/Experimental procedures

### Tissue samples

A total of six (*n* = 4) metastatic high-grade serous ovarian carcinomas omental tissues and four (*n* = 4) normal non-metastatic omentum tissues were obtained from patients who underwent surgery for benign conditions or cytoreductive surgery at King Faisal Specialist Hospital and Research Center. The institutional review board has approved the acquisition of tissue specimens and patient clinical information and all patients’ consent was duly obtained before the collection of samples (RAC#2170034).

### Reagents, cell lines, and cell culture

Ovarian cancer cell lines OV2774, SKOV3, OVCAR3, and HEK293T were obtained from the American Type Culture Collection (ATCC, USA). The highly metastatic SKOV3-ip variant was derived from parental SKOV3 cells from ascites arising in a nude mouse given an i.p. injection. All cells were cultured in DMEM/F12 medium supplemented with 10% fetal bovine serum (FBS; Invitrogen, USA). For the transwell coculture model, OvCa cells were seeded on the lower chamber of the 6-well plate, and a culture insert with 0.4 μm pore size (Corning, USA) was placed on the top of each well followed by seeding 2 × 10^5^ mesothelial cells in the upper chamber of the transwell.

### Cell viability assay

OvCa cells were seeded in 96 well plates were treated with cisplatin, paclitaxel, or doxorubicin. After 48 hours, cell viability was analyzed by the WST-1 (Sigma, USA) assay. The percentage of cell viability was expressed as relative to that of untreated control in each group.

### Cell labeling with DiD dye

Cells were stained with PHK26 dye (Invitrogen, USA) according to the manufacturer’s protocol. Briefly, 1 × 10^6^ cells/ml were incubated with PHK26 dye (0.5 μM) in serum-free medium at 37 °C for 45 minutes, washed with serum-free medium three times, and resuspended with PBS.

### Primary mesothelial cells isolation and culture

Human primary omental mesothelial and fibroblast cells were obtained from patients undergoing surgery for either benign peritoneal effusion or from primary tumor tissues or omental mesothelium containing metastatic tumors. The purification of mesothelial cells was performed as previously described [3] and was confirmed by positive staining of >99% calretinin (a mesothelial cell marker) by immunofluorescence. The 3D omental culture system was designed by plating 5000 human primary fibroblast cells per well and 0.5 μg of collagen type 1 [3]. After 45 minutes, human mesothelial cells were plated at a density of 20,000 cells.

### Primary omental mesothelial - OvCa cells coculture system

Human primary omental mesothelial cells were seeded at 2 × 10^5^ cells/well in a 24-well plate. At 80% confluency, luciferase/GFP+ (GFP-labeled) control and Jag2OE cells were added at 1 × 10^5^ cells/well in triplicate and treated with DMSO or MRK-003 (1 μM). The spent medium supplemented with drugs was changed every 3-days. After 1-week of co-culture, cells were subjected to a luciferase assay to selectively quantify the number of tumor cells. The values were normalized against luciferase quantification seeded with tumor cells alone. For gene expression analysis, primary human mesothelial cells were grown to 80% confluency in a cell culture plate. GFP-labeled control (2 × 10^5^ cells) and Jag2OE cells were seeded onto the plates in mesothelial cell-derived media. After 5 days in co-culture, cells were sorted to collect the GFP-negative mesothelial cells.

### Tumor xenografts and mouse omentectomy model

All procedures pertinent to mice and related experimental protocols were approved by The King Faisal Specialist Hospital and Research Center Animal Care and Use Committee (RAC#2170034). For omental metastatic studies, 1 × 10^6^ tumor cells were injected i.p. in female athymic nu/nu mice, and the development of metastasis/tumor burden was assessed by weighing the mice every 3 days and comparing the mice weight with the initial weight and at the end of the experimental endpoint before sacrificing the animals. Omental metastasis-free survival represents the time points at which each mouse developed omental metastasis by the difference in mouse weight. For omentectomy surgery on mice, general anesthesia protocols were followed. Briefly, laparotomy was performed through a 1-cm incision in the region of the stomach, and the omentum was carefully removed. The abdominal wall was then closed in two layers with a fine surgical suture. The sham surgery was performed as described above with the omentum carefully lifted before being placed back in the abdominal cavity. Once mice recovered from surgery-related trauma (1 week after surgery), 1 × 10^6^ cells were injected i.p. in 300 μl of sterile PBS. Tumor burden was monitored as described above. To establish the co-injected xenograft model, 1 × 10^6^ cells were mixed with or without 1 × 10^6^ mesothelial cells immediately before injection. Cell suspension was injected subcutaneously into mice. For cisplatin treatment, cisplatin is administered intraperitoneally at 5 mg/Kg body weight twice a week for a total of 3 weeks. Tumor size was measured every 2 and 3 days and tumor volumes were recorded.

### Limiting dilution assay (In vivo)

For the HPOMC co-culture model, tumor cells were monocultured or co-cultured with HPOMC cells for 10 days, and 1000, 5000, and 10,000 tumor cells suspension were injected subcutaneously in female athymic nu/nu mice in PBS. Mice were monitored every week for the appearance of tumor growth.

### Sphere formation assay

OV2774 and SKOV3 single cells were cultured in 6-well ultra-low attachment plates at a density of 5000 viable cells/well, supplemented with 0.4% BSA, 1% penicillin and streptomycin, B27, 20 ng/ml hEGF, 5 μg/ml insulin, 20 ng/ml FGF, 50 ng/ml hydrocortisone and 4 μg/ml heparin. Spheres were treated with cisplatin, eugenol, and a combination of both. The number and size of spheres were viewed under the microscope and counted the number of spheres (> 50 μm) every 3 days.

### Ex-vivo human omentum culture

For the ex-vivo omental culture experiment, a fresh surgically removed full human omentum was cut into small pieces with equivalent weight. All omental pieces were placed on a 24-well plate. The omentum pieces were cultured with 1 × 10^6^ cells and incubated at 37 °C for 2 days. Next, omental pieces were digested with 0.2% trypsin-EDTA for 10 minutes at 37 °C and scraped with a spatula. The trypsin was deactivated, and collected cells were pelleted. For the analysis of Jagged2 expression, fluorescently labeled OvCa cells and omental surface cells were sorted by FACS (Fluorescence activated cell sorting).

### Invasion assays

Jag2OE OV2774 or control cells were resuspended at 1 × 10^6^ cells in a serum-free medium and placed in an 8-μm-pore transwell (Corning, USA) with Matrigel. The top inserts were placed in wells that contained media with serum (10% FBS). 24 hours after seeding cells in inserts, serum-containing media was aspirated, and 500 μL 0.2% trypsin-EDTA was added to the wells to trypsinize the cells that had passed through the 0.8 μm pores. Trypsin activity was neutralized with a complete serum-containing medium and then cells were pelleted by 5-minute centrifugation. The cell pellet was resuspended with 1 ml of complete medium and the number of invaded cells was counted.

### Gene set enrichment analysis

For gene set enrichment analysis, we used GSEA v4.1.0 [17]. We used a normalized large panel of microarray expression data [18] of normal and high-grade metastatic and non-metastatic ovarian cancer patients’ tumors that were ranked ordered by expression using the provided signal-to-noise metric. TGF-β-responsive gene sets were generated by considering the top 50 genes from TGF-β-response signatures among the metastatic and non-metastatic groups. The upregulated pathways were defined by a normalized enrichment score (NES) > 0 and down-regulated pathways were defined by an NES < 0. Finally, gene sets were tested for enrichment in a rank-ordered list *via* GSEA using weighted statistics and compared to enrichment results from 1000 random permutations of the gene set to obtain *P*-values. Pathways with an FDR-*P*-value < 0.05 were chosen as significantly enriched.

### Notch reporter, Hes1-siRNA, and Smad3-siRNA transfection assays

For mesothelial cell transfection experiments, cells were seeded at 1 × 10^5^ cells/well in a 12-well plate and grown until the cell reached 80% confluency. For reporter assay, the firefly luciferase Notch reporter (BPS Bioscience, USA) and/or Renilla luciferase control vector (Promega, USA) plasmids were transfected using Lipofectamine 2000 at the concentration described by the manufacturer’s instructions. After 24 hours, the transfection medium was changed to a regular growth medium containing 1 × 10^5^ vector control or Jag2OE tumor cells/well and plated in triplicate in the presence of DMSO or MRK-003. After two days, the coculture was lysed and subjected to a luciferase assay in which firefly counts (Notch reporter activity) were divided by renilla counts to normalize for transfection efficiency. Mesothelial cells were transfected with scrambled or Hes1 and Smad3 siRNAs (cat#4390828; Ambion, USA) using Lipofectamine 2000 following the manufacturer’s instructions. After 24 hours, the transfection medium was changed to a regular growth medium. After 1 week, the coculture was lysed and subjected to luciferase assay to selectively quantify the number of tumor cells.

### RNA from GFP-labeled omental culture or primary human mesothelial cells

GFP-labeled OvCa cells were cocultured on freshly resected full human omentum, in a 3D omental culture, or primary human mesothelial cells. After co-culture, cells were sorted by FACS in PBS. This FACS sorting procedure separated labeled OvCa cells from mesothelial cells after co-culture. A lysis buffer was used to isolate RNA as described above.

### Establishment of stable knockdown and overexpression cells

JAG2 (cat#TL303860) human 4-unique shRNA constructs in lentiviral GFP vector were purchased from Origene (OriGene Technologies, USA). Stable shRNA-mediated JAG2 knockdown was obtained with the pGFP-C-shLenti and pGFP-V-RS plasmid vector system. shRNA vectors were inserted into 293 T packaging cells. After 48 hours viruses were collected, filtered, and used to infect target cells in 4 μg/mL polybrene. For stable overexpression of JAG2 human tagged ORF clone in human OV2774 OvCa cell line, packaging cells (293 T cell line) were transiently transfected with JAG2 or empty pLenti-C-mGFP-P2A-Puro vector using Lipofectamine 2000 (Invitrogen). The viral particle/supernatants were harvested 48 hours later and passed through a 0.45 μm filter. The filtered viral supernatant was resuspended in 4 μg/mL polybrene and added to the desired target cell cultures. Twenty-four hours after infection, cells were harvested and used for assays. Control cells contain the parental lentivirus vectors alone. The infected cells were selected and maintained in a 2 μg/ml puromycin-containing medium.

### Quantitative RT-PCR

Total RNA was extracted with a RNeasy mini kit (Qiagen, USA). cDNA was synthesized using superscripts III First-Strand (Invitrogen, USA). After reverse transcription, quantitative qRT-PCR was performed using the SYBR Green PCR master mix (Applied Biosystems, USA). Values were normalized against GAPDH in each sample. The reactions were run in triplicate. Relative levels of mRNA gene expression were calculated using the 2^ΔΔCT^ methods. Differences between treatments were evaluated using an unpaired two-tailed Student’s *t*-test. Supplementary Table S1 contains information on the primers used in this study.

### Immunofluorescence and Image Analysis

The immunofluorescence staining was performed as described previously [19]. In short, cells were cultured on a glass coverslip, washed with PBS, and fixed in ice cold methanol. Permeabilized with 0.1% Triton X for 3 minutes, blocked with 5% BSA for thirty minutes. Afterwards incubated with primary antibodies [1;100, Sigma, USA] overnight at 4 °C. Finally, washed with PBS and blocked secondary antibody and mounted the coverslips with mounting medium (Vecta Shield, USA) with DAPI (4,6-diamidino-2-phenylindole).

### Immunoblot analysis

Cells were lysed with SDS lysis buffer, and an equal amount of heat-denatured proteins was loaded in each blot, separated on an SDS-PAGE gel, and transferred to a PVDF membrane. Once transferred, the membrane was blocked with 5% milk. The following antibodies and dilutions were applied overnight at 4 °C: rabbit anti-Jag2 (1: 1000, cat#MA5-37895; Thermo Fischer Scientific, USA), rabbit anti-Smad3 (1:1000, sc-101154, Santa Cruz, USA), rabbit anti-phosphoSmad3 (1:1000, sc-517575, Santa Cruz, USA), rabbit anti-GAPDH (1:1000, sc-32233, Santa Cruz, USA) for loading control. The blots were incubated with horse radish peroxidase-conjugated anti-rabbit secondary antibody for an hour at room temperature and visualized with enhanced chemiluminescence detection reagents.

### Human IL-6 ELISA assay

Human IL-6 in the conditioned medium of cultured or cocultured cells were quantified in triplicate using an ELISA kit (Human ELISA kit, Abcam, USA, cat# ab-178013).

### Pharmacological inhibitor MRK-003

The preclinical use, pharmacokinetics, and pharmacodynamics of MRK-003 (Merck Research Laboratories) have been well-reported previously [20]. In our in vivo experiments, mice were given 0.5 percent methylcellulose as a vehicle or MRK-003 at a dose of 100 mg/Kg, both freshly prepared before usage. Before administering each dose, the MRK-003 was well mixed to ensure that it was uniformly dispersed in the solution. The MKR-003 dosing cycle consisted of two days on and five days off. For in vitro experiments, MRK-003 was dissolved in DMSO and was administered at 1 and/or 5 μM.

### Cell cycle analysis

Cell cycle analysis was carried out using the Cell Cycle Detection Kit (Invitrogen, USA) following the manufacturer’s instructions after the cells had been co-cultivated with mesothelial cells in a transwell plate, cultured with the conditioned media, or treated with DMSO or MRK-003. Trypsinization was used to collect OvCa cells, which were then washed in ice-cold PBS and fixed in a 75% ice-cold ethanol solution. Before labeling, cells were gently resuspended in ice-cold PBS. Next, propidium iodide (PI) was incubated with the cells for 30 minutes at room temperature. Following that, data were examined by NovoExpress software and the Novo Express flow cytometer (Agilent, USA).

### Neutralizing inhibitor and recombinant proteins

TGF-β-receptor 1 (EMD616451; EMD Bioscience, USA) was resuspended in DMSO. For in vitro studies, OvCa tumor cells were seeded on a 6-well plate and treated with either DMSO or EMD616451. Cells were then treated with recombinant TGF-β1 (R & D Systems, USA) was dissolved in PBS, and administered at a concentration of 50 pM for various time points. Proteins and RNA were collected and analyzed for gene expression by qRT-PCR as described above. Recombinant human IL-6 (R & D Systems, USA) was dissolved in sterile PBS containing 0.1% FBS and administered at 10 and 100 ng/mL concentrations.

### Statistical analysis

All statistical analysis was performed using R-Statistical (version 4.0.3) software. All graphs were generated from the R-package “ggplot2”. The significance of data in vitro and in vivo assays was assessed by unpaired or paired (where indicated) two-tailed Student *t*-test. Results are presented as average +/- standard deviation (SD) or as average +/- standard error of the mean (SEM). The significance of data between groups was assessed by Mann-Whitney *U*-test or ANOVA. Comparisons between Kaplan-Meier curves were performed using the log-rank test from R-packages “survival” and “survminer”. *P*-values of < 0.05 were considered significant.

## Results

### Mesothelial cells from omentum promote OvCa cell growth, invasion, and chemoresistance

**Fig. 1:**
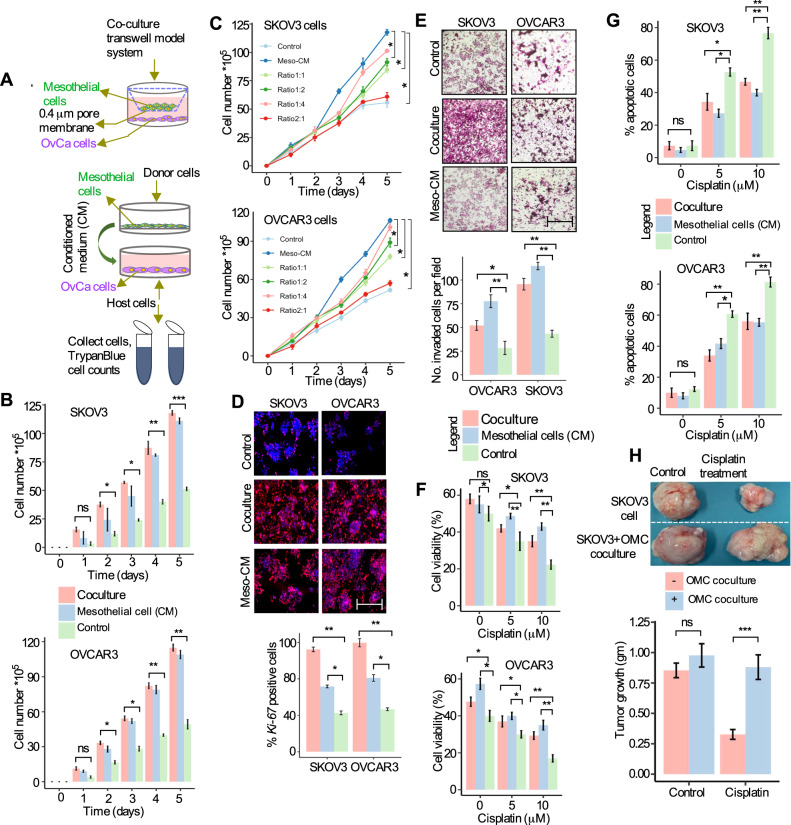
Mesothelial cells induce the growth of OvCa cells. **A** A schematic model of OvCa cells cocultured with primary mesothelial cells in a transwell plate (co-culture, top) or cultured in mesothelial cells-conditioned media (CM) (bottom). **B** SKOV3 and OVCAR3 cells were co-cultured with mesothelial cells or incubated with mesothelial cell-derived conditioned medium (Meso-CM) in the co-culture transwell system for the indicated time periods followed by trypan blue staining and counting cells. SKOV3 and OVCAR3 cells alone served as control. Error bar represent Mean +/- SD; ns - not significant; *P < 0.05, **P < 0.01, ***P < 0.001 by ANOVA for repeated measures. **C** OvCa cells were cultured in the conditioned medium with a different ratio of culture medium and supernatants from mesothelial cells (ratios 1:1, 1:2, 1:4, 2:1, and total supernatants from the mesothelial cell culture, respectively) followed by quantitation of cells. Error bar represent Mean SD + /-, *P < 0.05 by repeated measures of ANOVA**. D** OVCAR3 and SKOV3 cells were used for immunofluorescence staining of *Ki-67* on day 5 (post-culture) in the indicated three groups. The bar graph below shows the quantification of the proportion of *Ki-67*-positive cells in each group. Scale bar 100 μm, Error bar represent Mean +/- SD; *P < 0.05, **P < 0.01 by one-way ANOVA. **E** Matrigel invasion assay of SKOV3 and OVCAR3 cells in the indicated three groups. Scale bar 100 μm, *n* = 3. The graph below represents the quantification of the number of invaded cells per field, Error bar represents Mean +/- SD; *P < 0.05, **P < 0.01 by one-way ANOVA. **F** Assessment of the effect of Meso-CM or mesothelial cell co-culture on the sensitivity of SKOV3 and OVCAR3 cells to cisplatin. Cell viability is normalized to the untreated control group and statistical analysis was compared with SKOV3 and OVCAR3 monoculture group (*n* = 3), Error bar represents Mean +/- SD; ns= not significant, *P < 0.05, **P < 0.01 by one-way ANOVA. **G** SKOV3 and OVCAR3 cells were treated with the indicated concentrations of cisplatin for 48 hours and the proportion of apoptotic cells was determined by Annexin V-PI flow cytometry. Quantification of percentages of apoptotic cells in the indicated groups. Each group was statistically analyzed and compared to the untreated control group (*n* = 3), Error bar represents Mean +/- SD; *P < 0.05, **P < 0.01 by one-way ANOVA. **H** Effects of mesothelial cell co-culture on cisplatin treatment resistance of SKOV3 cells in vivo. SKOV3 cells were either cultured alone or were co-cultured with human mesothelial cells in vitro, and then were injected subcutaneously into immunocompromised mice. Once tumors were palpable, mice were treated with cisplatin (5 mg/kg body weight) or DMSO twice a week for 3 cycles. Representative of resected tumor images and tumor weight (bar graph) at the end point is shown (*n* = 1, 2, 3 mice per group). ns = not significant; ***P < 0.01 by two-way ANOVA. Data in the figure represent average and +/-SEM; *P*- values were determined using the Student’s *t*-test unless otherwise indicated.

The omentum is the most prevalent location of ovarian cancer metastases in women with serous high-grade ovarian cancer, which is composed of mesothelial cells that secrete fibronectin in the presence of OvCa cells and promote tumor growth [3]. These findings imply that the omentum contributes to tumor cell proliferation and invasion in the omentum, which enables tumor cells to colonize the tumor-stromal microenvironment niche during the early metastatic stage. Therefore, we started the present investigation by exploring whether the growth of OvCa cells is induced when co-cultured with primary mesothelial cells and to determine the role of omental mesothelial cells in OvCa cell growth and colonization. The microscopical analysis allowed us to identify the metastasis-free or disease-free omentum, from which we were able to separate primary mesothelial cells. In order to confirm that the cells were mesothelial, human primary omental mesothelial cells (henceforth referred to as HPOMC) were grown in vitro, and stained with the calretinin-specific mesothelial cell marker (Figs. S1A, B). We next co-cultured OvCa cells (SKOV3 and OVACR3 cells) with HPOMCs on a transwell plate for five days (Fig. 1A). When OvCa cells were co-cultured with HPOMCs, their numbers increased significantly (Fig. 1B-C). In concordance, the number of OvCa cells was increased when cultured in the conditioned medium (CM) derived from HPOMCs cultures (Fig. 1A, C, Figs. S1C, S1D, Table S2). Furthermore, when co-cultured with HPOMCs or conditioned medium from HPOMCs (HPOMC-CM), the number of *Ki-67* positive OvCa cells and several key cell cycle genes (CCND1, CDK1, CDK2, and RB) were also considerably elevated (Fig. 1D; Figs. S1E, S1F, Table S2). This demonstrates the role of HPOMCs in enhancing OvCa cell growth.

We have then investigated whether conditioned media from OvCa-mesothelial cell cultures or direct co-cultures of OvCa cells with mesothelial cells can enhance OvCa cell invasion using Matrigel-coated inserts primed with conditioned media from OvCa-mesothelial cells or co-culture. Figure 1E shows that HPOMC-CM co-cultured OvCa cells significantly enhanced the OvCa cell invasion compared to control cells. These findings imply that co-cultures of tumor and mesothelial cells or HPOMC-CM-conditioned medium can enhance the proliferative and invasive capabilities of OvCa cells in vitro.

Next, we assessed the cisplatin sensitivity of OvCa cells grown with HPOMC-CM or OvCa cells directly co-cultured with HPOMC cells. Cisplatin sensitivity of OvCa cells was decreased when co-cultured with HPOMC cells or HPOMC-CM (Fig. 1F). Consequently, cisplatin-induced cell death in OvCa cells was also significantly reduced as compared to controls (Fig. 1G). Given that the majority of OvCa patients exhibit multidrug resistance, we examined chemosensitivity with two additional chemotherapeutic drugs. HPOMCs reduced the sensitivity of OvCa cells to paclitaxel and doxorubicin, (Figs. S1G–J). The next step was to see if HPOMCs obtained from individuals without omental metastases had comparable antitumor effects on OvCa cells. OvCa cells co-cultured with HPOMCs or omental metastatic mesothelial cells (OMMCs) had no discernible impact on the OvCa cells’ susceptibility to cisplatin (Figs. S1K-M). To confirm these results, we have evaluated the impact of HPOMCs on OvCa cells’ sensitivity to cisplatin in tumor xenografts. To this end, SKOV3 cells (a human ovarian cancer cell line obtained from ascites) were first either cultured alone or co-cultured with freshly isolated HPOMC cells, and then were injected into mice. When tumors reached reasonable volumes, animals were treated with or without cisplatin every three days for 3 cycles. In contrast to tumors formed with SKOV3 cells grown without HPOMC cells, tumors formed with co-cultured cells showed resistance to cisplatin (Fig. 1H). These findings indicate that mesothelial cells derived from omentum promote the chemoresistance capacities of OvCa cells.

### Ovarian cancer cells secret the Notch ligand Jagged2 to promote tumor growth in the omentum

**Fig. 2:**
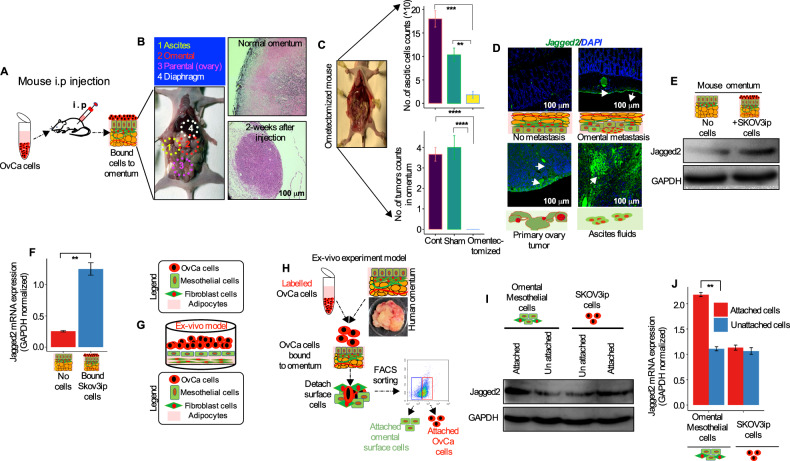
OvCa cells induce Jagged2 expression early in metastasis to the omentum. **A** Schematic diagram of SKOV3-ip cell injection in mouse peritoneum. **B** Locations of (i) ascites, (ii) omentum, (iii) primary ovary, and (iv) diaphragm tumor progression 3- weeks after i.p. injection (left panel). H & E staining of omentum without (top right panel) and with tumor (right bottom panel). **C** Endpoint analysis of malignant ascites and total number of tumor nodules in omentectomized mice 4 weeks after intraperitoneal cell injection of SKOV3-ip cells. Data are shown as Mean +/-SEM (*n* = 3). Statistical significance was calculated using Kruskal-Wallis one-way ANOVA followed by Dunn’s multiple comparison test. **P < 0.01, ****P < 0.0001. **D–F** Jagged2 protein and RNA expression in whole mouse omentum three weeks following in vivo i.p. injection of SKOV3-ip tumor cells. Immunofluorescence (**D**), Immunoblot (**E**), and qRT-PCR (F) analysis were performed using Jagged2 specific antibody and probe (**P < 0.01; Scale bars 100 μm). **G** Diagram of the mesothelium in 3D culture. Primary human omental fibroblasts are plated with human omental ECM and cultured for 6 hours. The fibroblasts are overlaid with human omental mesothelial cells and cultured for an additional 24 hours before OvCa cells are seeded. The purpose of the use of the 3D culture system was to investigate OvCa cells’ adhesion, invasion, and proliferation. **H** Ex-vivo human omentum adhesion, invasion, and proliferation assay with PHK25 labeled OvCa cells and the schematic of cell sorting by FACS. **I** Western blot analysis of Jagged2 protein in the surface cells of the human omentum in two culture conditions. Cells were cultured in human omentum without fluorescently labeled cells (unattached) or PHK25 labeled SKOV3-ip cells (attached) after FACS sorting. **J** qRT-PCR mRNA analysis of Jagged2 following the same methods described in H (**P < 0.01).

Bidirectional communication and nutrient exchange between cancer cells and stromal cells in the tumor microenvironment (TME) appears to be a key step in promoting metastatic tumor development [21]. The peritoneal dissemination of OvCa has been mimicked in mice using numerous cell lines, including ID8 and SKOV3 [3, 22, 23]. Thereby, we employed the SKOV3-ip cell line to track the tumor growth and progression following intraperitoneal (i.p.) injection (Fig. 2A). Two weeks later, we spotted tumor cells throughout the peritoneal cavity, including the omentum, primary ovary site, and diaphragm (Fig. 2B). In humans, the omentum is an adipose tissue created from a fold of the peritoneal mesothelium that covers the greater omentum in the majority of the abdomen, while in mice it is merely a narrow stretch of adipose tissue found between the stomach, pancreas, and spleen [23] (Fig. 2B). We transplanted SKOV3-ip cells into omentectomized mice (for details in removing omentum, please refer to materials and methods section) and tracked tumor development for 4-weeks following i.p. injection to determine the requirements of the omentum and omental metastasis. Mice-bearing cells with nonomentectomized (control) and the sham-operated group showed a comparable course of tumor growth and progression with the accumulation of ascitic and omental tumors after 7–10 days (Fig. 2C). On the other hand, tumor growth is absent in omentectomized mice, and the near complete absence of ascitic cells is the evidence that the omentum is a crucial premetastatic niche for tumor growth and progression (Fig. 2C). The histological investigation in mice omentum demonstrated tumor cell infiltration into the omentum (Fig. 2B, C), supporting the idea of a tumor cell’s ability to implant into the omentum. We have next investigated if Jagged2 expression in metastatic OvCa is functionally significant, and whether early OvCa cell metastasis could be mimicked in vivo (Fig. 2A) and ex vivo (Fig. 2G, H) with human omental tissue. Following the i.p. injection of OvCa cells, immunofluorescence labeling revealed a significant increase in Jagged2 expression in the mouse omentum (Fig. 2D). Additionally, the levels of the Jagged2 protein and mRNA were lower in OvCa cell-free omentum (Fig. 2B, D-F). In an ex-vivo model, OvCa cells were fluorescently leveled to distinguish them from HPOMCs, and then overlaid in a freshly excised human omentum for 24 hours (Fig. 2G, H). We found that the Jagged2 protein and mRNA levels were considerably higher in the PHK25-labeled surface cells that had interacted with cancer cells compared to controls, whereas the expression of Jagged2 in the cancer cells remained unaltered (Fig. 2I, J). Collectively, our results suggest that tumor cell-derived Jagged2 promotes ovarian cancer metastatic tumor growth in the omentum.

### Notch ligand Jagged2 is associated with a high risk of omental metastasis

**Fig. 3:**
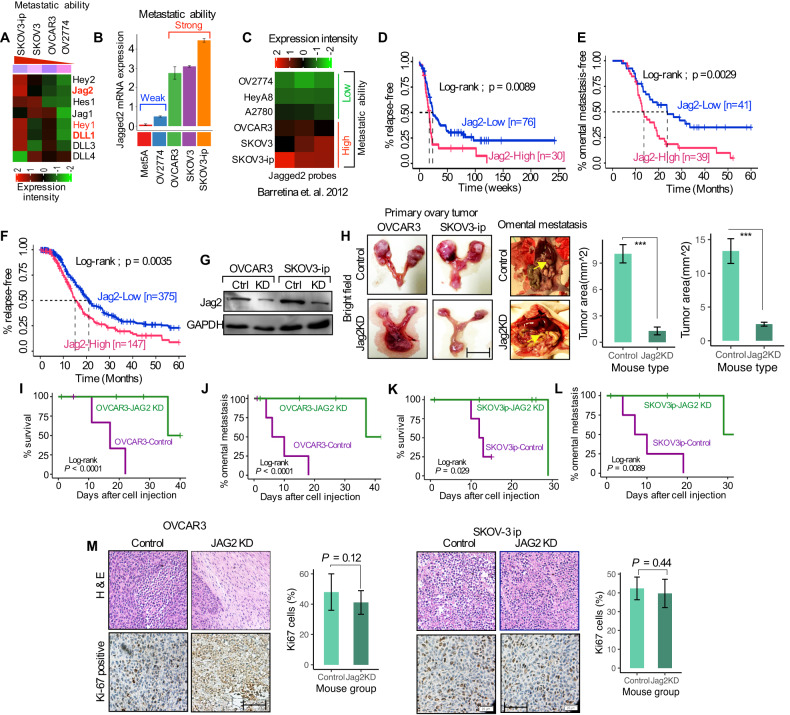
Notch ligand Jagged2 is associated with a high risk of omental metastasis. **A** Heat map showing the qRT-PCR gene expression of the Notch pathway ligands and downstream targets in four ovarian cancer cell lines. **B** qRT-PCR analysis of the Jagged2 gene expression in ovarian cancer cell lines with distinct metastatic abilities. **C** Heat map showing the microarray data analysis [30] of the Jagged2 mRNA expression in ovarian cancer cell lines with metastatic ability. **D**–**F** Kaplan-Meier progression-free survival curves of ovarian cancer patients from Gentric (GSE26193) [31], Denkert (GSE14764) [32], and TCGA [33] data sets representing high and low expression of Jagged2. **G** Western blot analysis of the Jagged2 protein in control and Jagged2 knockdown (KD) in OVCAR3 and SKOV3-ip cells. **H** (Top) Representative images of tumor nodules in mouse metastatic omentum (right) and tumors developed in the primary ovary (left). (Bottom) quantification of tumor area from each group. Scale bar 100 μm. ***P < 0.001. **I–L** Kaplan-Meier survival and omental metastasis- free curves of mice injected with control or Jagged2 knockdown (Jag2KD) OVCAR3 and SKOV3-ip cells (n = 5 mice per/group). **M** (Left panel) Representative immunohistochemical images of *Ki-67* and H & E histological images of primary omentum from representative mice for each experimental group. (Right panel) quantification of *Ki-67*-positive cells in the Jag2KD cells compared with the control group. Scale bar 20 μm. *P* = 0.12 (OVACR3 cells) and 0.44 (SKOV3-ip cells). Data in the figure represent average and +/-SEM; *P*-values were determined using the Student’s *t*-test unless otherwise indicated.

Recent genomic profiling of OvCa revealed that the Notch pathway alterations are among the most prevalent genomic changes [24, 25]. Given the importance of Notch signaling in ovarian cancer development and adhesion [12], we first investigated the endogenous expression of the Notch ligands, receptors, and several downstream targets in a series of OvCa cell lines with the potential of metastatic abilities [26, 27]. Notably, all the OvCa cell lines that form primary tumors in mice had identical growth patterns. However, SKOV3 and SKOV3-ip cells are capable of developing omental metastases [28, 29]. We examined gene expression of the Notch pathway receptors, and several notable downstream Notch target genes, which showed the least to moderate associations with the metastatic ability (Figs. S2A, S2B), nonetheless, the Notch ligands Hey1, Hes1, DLL1, and Jagged2 levels were noticeably increased in the SKOV3-ip cells (Fig. 3A; Fig. S2C). Additionally, the expression of the Jagged2 gene in OvCa cell lines revealed that SKOV3 and SKOV3-ip cells with aggressive metastatic ability expressed high levels of Jagged2 (Fig. 3B, C; ref. [30]). These findings point to Jagged2 as a clinically significant driver and suggest a possible link to OvCa omental metastasis.

The clinical importance of Jagged2 expression in OvCa metastases was then assessed using previously reported data sets. The Gentric [31] and Denkert [32] datasets revealed that the expression of Jagged2 was significantly higher in patients with relapse (Log-rank *P* = 0.0089; *P* = 0.0029), and the incidence of relapse was significantly linked with the Jagged2 expression level (Fig. 3D, E). Compared to the Gentric and Denkert data sets, the TCGA data set [33] differs in that it includes more patient sample data and a wider variety of clinical criteria. The TCGA data showed that patients with high Jagged2 expression exhibited considerably more OvCa metastases than patients with low Jagged2 expression (Fig. 3F; log-rank *P* = 0.0035). The incidence of progression-free survival, however, did not differ substantially amongst patients with differing Notch ligands and Hes1 expression (Figs. S2D-S2H). These results further support the notion that Jagged2 plays an important role in OvCa omental disease progression, in contrast to Notch receptors or other Notch pathway components.

To assess the functional role of Jagged2 for ovarian cancer cells’ capacity to undergo omental metastasis, we stably knocked down Jagged2 in two metastatic OVCAR3 and SKOV3-ip cell lines (Fig. 3G), which express high levels of Jagged2 (Fig. 3B, C). Following the i.p injection of tumor cells, the formation of tumors and the progression of omental metastasis were monitored. Jagged2 knockdown dramatically reduced omental metastasis of tumor cells in the mouse omentum (Fig. 3H), with an improvement in survival (Fig. 3I, K), and a marked delay in the initiation of omental metastasis in mice (Fig. 3J, L). In OVCAR3 and SCOV3-ip cells, the average weight of primary tumors was approximately 0.81 gm, and 0.95 gm, while average ascites volume was 4.14 mL and 4.61 mL (Table 1). Additionally, disseminated tumor nodules were also scraped off and isolated from the cavity for further analysis, and their weighs ranged from 1.56 g to 2.16 g, respectively (Table 1). Furthermore, the number of *Ki-67* positive cells in OVCAR3 and SKOV3-ip remained unaltered following Jagged2 knockdown (Fig. 3M), indicating that Jagged2-deficient cells might grow during omental metastasis. Importantly, these findings imply that tumor-derived Jagged2 has a functional role in omental metastasis, primarily through creating and sustaining a robust microenvironmental niche.

**Table 1:** Tumor burden and ascites volume in mice injected with OVCAR3 and SKOV3-ip human ovarian cancer cell.

Xenograft model	OVCAR3 (*n* = 5)	SKOV3-ip (*n* = 5)
Primary tumor (g)	0.87 + /− 0.22	0.95 + /− 0.31
Disseminated tumor (g)	1.56 + /− 0.16	2.13 + /− 0.35
Ascites (mL)	4.14 + /− 1.05	4.61 + /− 1.16

### Ectopic expression of Jagged2 in ovarian cancer cells promotes omental metastatic tumor growth

**Fig. 4:**
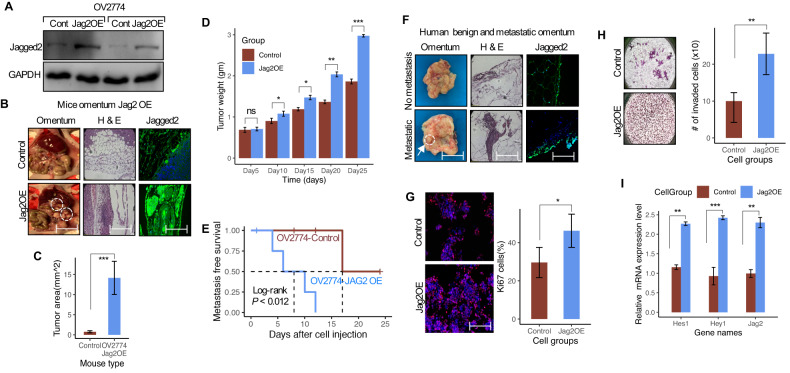
Enforced expression of Jagged2 in ovarian cancer promotes omental metastasis. **A** Western blot analysis showing Jagged2 protein expression in control and Jag2OE OV2774 cell line. **B** Mice inoculated with control and Jag2OE cells showed the absence (*n* = 5) and presence (*n* = 5) of omental tumor nodules with histological and Jagged2 immunofluorescence. Magnification of images are taken at 40x (scale bar = 200 μm). **C** Quantification of tumor area in the omentum from each mice group (*P* = 0 .0019). Error bar represent Mean +/- SD; ***P < 0.001. **D** Quantification of tumor weight (in grams) from each mice group from the day of inoculation (0 days) up to day 25. Error bar represent Mean +/- SD; ns - not significant; *P < 0.05; **P < 0.01 and ***P < 0.001, respectively. **E** Kaplan-Meier omental metastasis-free survival curve of mice inoculated with control (*n* = 5) or Jag2OE (*n* = 5) (Log-rank *P* < 0.012). **F** Representative images of freshly resected human omentum showing absence (no omental metastasis; *n* = 4) and presence (omental metastasis; n = 4) of omental tumors with histological analysis of both H & E and Jagged2 immunofluorescence. Scale bar 100 μm. **G** Immunofluorescence staining of *Ki-67* from omental metastatic tumors from each group (left panel). The right panel illustrates the quantification of the percent of *Ki-67*-positive tumor cells. For the quantification analysis, 10 distinct regions were randomly selected from each group. *P < 0.05. **H** (Left panel) Representative images of invaded cells and (right panel) quantification of cells invasive capability of control and Jag2OE cells using two-chamber transwell Matrigel invasion assay. **P < 0.01. Scale bar 100 μm, **I** qRT-PCR mRNA expression analysis of the Notch target genes Hes1 (**P < 0.01), Hey1 (***P < 0.001), and the Notch receptor Jagged2 (**P < 0.01) in control and Jag2OE OV2774 mice omental metastatic site. Data in the figure represent average and +/-SEM; *P*-values were determined using the Student’s *t*-test unless otherwise indicated.

To confirm the role of Jagged2 in OvCa omental metastasis, we overexpressed the Jagged2 gene in the poorly metastatic OV2774 cells (Fig. 4A). Following i.p. injection, mice bearing tumors with Jagged2 overexpression (Jag2OE) showed implants on the omentum (Fig. 4B). To further analyze the omental metastasis, mice omental tissues were isolated and stained for Jagged2. Jag2OE cells were largely found at the onset of tumor cell spread, accompanied by an increasing tumor area and weight in the omentum (Fig. 4B-D). Furthermore, Jag2OE tumor cells developed omental metastases earlier than the control (only one mouse in the control group developed small foci of tumor; Fig. 4E). Our clinical sample examination of omental tissues from patients with metastatic ovarian cancer revealed a similar observation. The expression of Jagged2 was first verified in surgically isolated benign (*n* = 4) and metastatic (*n* = 4) omental samples from a human with ovarian cancer. In comparison to patients without metastasis, all metastatic patients had stronger levels of Jagged2 expression in both the stroma and the periphery of the omentum (Fig. 4F). This indicates that tumor-derived Jagged2 cells can establish tumors preferentially in the omentum. Furthermore, the Jag2OE group had a greater number and statistically significant proportion of *Ki-67*-positive cells in the omentum (Fig. 4G). Moreover, Jag2OE cells demonstrated the highest in vitro invasive potential (Fig. 4H). Most notably, we found that the expression of the Notch pathway target genes was increased in Jag2OE cells in tumor-associated omental mesothelial cells (Fig. 4I). These findings indicate that enforced expression of Jagged2 in low metastatic cells is sufficient to drive omental metastasis, possibly by activating the Notch pathway in the supporting omental microenvironment.

### Active TGF-β-Smad3 signaling pathway transcriptionally regulates Jagged2 during ovarian cancer omental metastasis

**Fig. 5:**
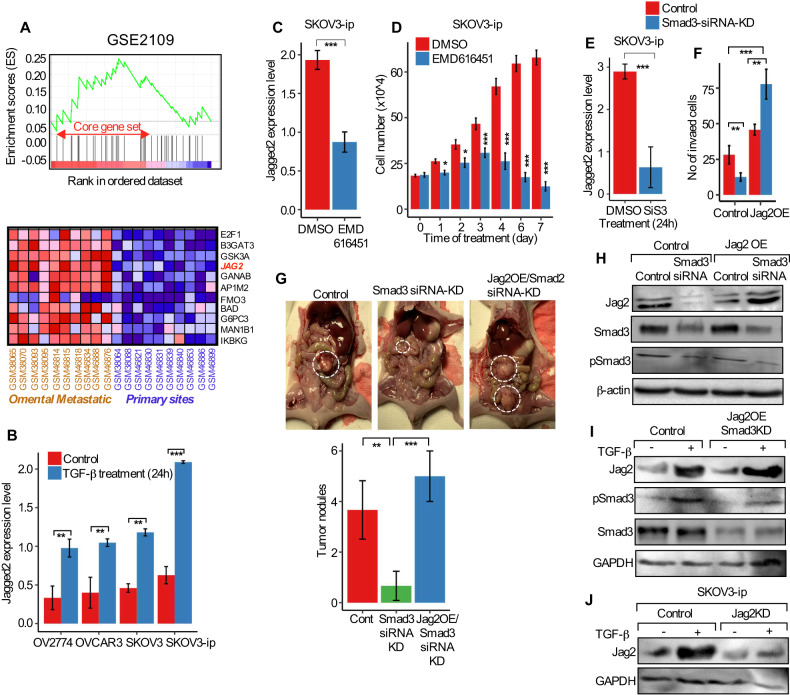
Jagged2 is functionally regulated by the TGF-β-Smad3 signaling pathway in ovarian cancer omental metastasis. **A** Gene-set enrichment analysis (GSEA) of the TGF-β-responsive genes set in a ranked list of differentially expressed genes in primary ovary tumors versus omental metastatic tumor cells (GSE2109; *P* = 0.037). The bottom panel shows a corresponding heat map of core TGF-β gene sets with elevated expression in two groups. **B** Jagged2 mRNA expression in response to TGF-β treatment in ovarian cancer cell lines. **P < 0.01, ***P < 0.001. **C** Jagged2 mRNA expression in SKOV3-ip cells treated with either DMSO or TGF-β-receptor inhibitor (EMD616451) for 24 hours. ***P < 0.001. **D** Cell proliferation assay of SKOV3-ip cells treated with either DMSO or TGF-β-receptor inhibitor (EMD616451) for the indicated time. *P < 0.05, ***P < 0.001. **E** Jagged2 mRNA expression in ovarian cancer cells treated with either DMSO or Smad3 inhibitor (SIS3) for 24 hours. ***P < 0.001. **F** Number of invaded cells in the indicated cells with altered expression of Jagged2 and Smad3. **P < 0.01, ***P < 0.001. **G** Representative images of the omental region in mice from experimental groups on day 10. (left) Quantification of tumor numbers in each group of experimental mice. Data in the figure represent average +/- SEM, SD. **P < 0.01, ***P < 0.001. **H** Western blot analysis showing Jagged2, Smad3, and phospho-Smad3 expression in the indicated cells with altered Jagged2 and Smad3. **I** Western blot analysis of Jagged2, phospho-Smad3, and Smad3 protein levels in the control or Smad3 knockdown in Jag2OE cells with or without stimulation of TGF-β. **J** Western blot analysis showing Jagged2 protein expression in the control and Jag2KD SKOV3-ip cells with or without TGF-β treatment. Data in the figure represent average and +/-SEM; *P*-values were determined using the Student’s *t*-test unless otherwise indicated.

The expression and influx of premetastatic genes have been reported to interact tightly with the tumor microenvironment and omental niche, which is predominantly regulated by distinct signaling molecules and plays a significant role in the development of metastatic tumors. Since omental mesothelial cells are the first point of contact for OvCa cells [3, 34], we sought to investigate the potential implication of the Jagged2 regulators in the omental microenvironment and the enrichment of multiple signaling pathways and target gene sets in the metastatic and non-metastatic ovarian tumor cells. Using the gene-set enrichment analysis (GSE2109), we have identified that the TGF-β pathway-responsive genes are considerably overrepresented among the upregulated genes in the metastatic omentum tumors compared to the primary site of disease (Fig. 5A, top panel *P* = 0.037). Importantly, Jagged2 was shown to be among the top 10-gene enrichment core of TGF-β-responsive genes (Fig. 5A, bottom panel), suggesting that Jagged2 is a possible target of TGF-β signaling in OvCa cells during omental metastatic tumor development. Because TGF-β1 is abundantly expressed and produced by omental mesothelial cells [35], we first examined whether mesothelial cells are able to regulate Jagged2. Interestingly, after 24 h of TGF-β1 stimulation, the Jagged2 mRNA was considerably increased in OvCa cell (Fig. 5B; Fig. S3A). On the other hand, treatment with TGF-β Receptor 1 kinase inhibitor (EMD-616451), prevented Jagged2 activation in OvCa cells (Fig. 5C; Figs. S3B, S3C) and decreased cell proliferation in vitro (Fig. 5D). Furthermore, Smad3 inhibition in OvCa cells, showed that Jagged2 is transcriptionally controlled by Smad-dependent TGF-β signaling (Fig. 5E-F).

Next, we have investigated whether Jagged2 is a key downstream effector of the pro-metastatic TGF-β-Smad signaling pathway during omental metastasis. We reasoned that if Jagged2 is an essential TGF-β target during omental metastasis, overexpression of Jagged2 in Smad3-KD cells would fully or partially restore the aggressive behavior of OvCa cells in vivo, influencing and initiating omental metastasis (Fig. 5G-I). Furthermore, decreased omental metastasis ability was seen in our Jag2KD experiments, which may indicate the Jag2KD cells’ inability to enhance Jagged2 expression in response to omental-derived TGF-β signaling (Fig. 5H; J). Collectively, our findings show that TGF-β, a well-known pro-metastatic cytokine, stimulated Jagged2 expression in OvCa cells, promoting metastatic dissemination and tumor growth in the omental premetastatic niche.

### OvCa cells expressing Jagged2 stimulate the Notch signaling in mesothelial cells and confer the growth advantage of tumor cells in the omental microenvironment

**Fig. 6:**
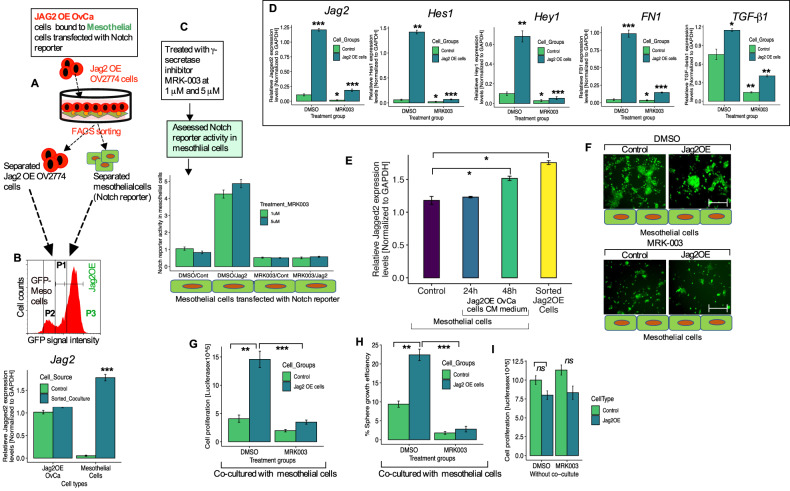
OvCa cells expressing Jagged2 have a growth advantage in the omental microenvironment *via* Notch-dependent cross-communication with mesothelial cells. **A** Schematic diagram of a co-culture model between Jag2OE tumor cells and human primary mesothelial cells transfected with a Notch reporter. **B** Flow cytometry cells separation of mesothelial cells (P2) from GFP+ Jag2OE cells (P3). After flow cytometry cell separation, the bottom panel shows the quantification of the Jagged2 mRNA expression control and sorted co-culture cells from the indicated group (***P < 0.001). **C** Co-culture between control or Jag2OE tumor cells and mesothelial cells transfected with a Notch reporter and treated with DMSO or MRK-003 (1 and 5 μM). **D** qRT-PCR assessment of the mRNA levels of the indicated Notch target genes, TGF-β1 and fibronectin 1 (FN1) in mesothelial cells separated by FACS from co-culture in each experimental group. *P < 0.05, **P < 0.01, and ***P < 0.001. **E** qRT-PCR analysis of Jagged2 mRNA expression in primary human mesothelial cells with or without a conditioned medium (CM) from Jog2OE cells for the indicated time periods. *P < 0.05. **F** Representative images of co-cultures of Jag2OE and mesothelial cells from each experimental group (DMSO or MRK-003 [5 μM]). Scale bar, 200 μm. **G** Quantifying tumor cell proliferation of control and Jag2OE cells after co-culture with mesothelial cells from each experimental group by luciferase assay. **P < 0.01, ***P < 0.001. **H** Quantification of sphere growth efficiency of control and Jag2OE after co-cultures of each experimental treatment group. **P < 0.01, ***P < 0.001. **I** Quantification of tumor cell proliferation cultured alone (no co-culture) from each experimental group. Data in the figure represent average and +/-SEM; *P*-values were determined using the Student’s *t*-test unless otherwise indicated. ns = not significant. All experiments were run in triplicates.

Manipulating Jagged2 expression in OvCa cells substantially impacted the development of omental metastatic performance without significantly affecting primary tumor functions. We, therefore, hypothesize that the Notch-Jagged2 signaling may enable cross-communication between tumor cells and the omental mesothelial cell milieu and promote a supportive environment for tumor cells to commence omental metastasis based on the evidence from earlier experiments (Figs. 4–5). It would be intriguing to determine how the Jagged2-Notch signaling facilitates this process more easier. To investigate the involvement of supporting mesothelial cells in Jagged2-mediated omental metastasis, we exploited a co-culture system (Fig. 6A) of Jag2OE OvCa cells with HPOMCs expressing a Notch reporter. We have also investigated the ability of tumor-derived Jagged2 to promote the Notch activity in mesothelial cells. We have found a 4-fold increase in the Notch activation in the HPOMCs (Fig. 6B). This increase was later abolished by the γ-secretase inhibitor (GSI) (MRK-003) (Fig. 6C). Furthermore, the co-culture of FACS-separated primary HPOMCs with GFP-labeled Jag2OE tumor cells resulted in a significant increase in the Jagged2 expression by the mesothelial cells (Fig. 6C). This was accompanied by the activation of many Notch pathway target genes, including fibronectin1 (FN1) and TGF-β1, whose expression was later inhibited with MRK-003 (Fig. 6D). This implies that when omental mesothelial cells interact with OvCa cells, transcriptional activation of the Notch pathway occurs. We subsequently investigated if an OvCa cell culture-conditioned medium might promote Jagged2 in mesothelial cells in the same manner. However, conditioned media (CM) derived from OvCa cells promoted Jagged2 expression in mesothelial cells in a time-dependent manner, indicating that Notch activity and activation of Jagged2 in primary mesothelial cells also occur as a result of indirect contact with OvCa cells (Fig. 6E). Furthermore, we found in that Jag2OE cells acquired ability for growth advantage during omental metastatic tumor growth. Given the distinct growth characteristics of Jag2OE cells in omental metastases, we investigated whether the growth advantage was acquired through cross-communication with mesothelial cells. By growing GFP-luciferase-labeled Jag2OE tumor cells on a monolayer of mesothelial cells, we evaluated and measured the tumor cell growth. Comparing the results to controls and those without mesothelial cell co-culture, it was shown that Jag2OE tumor cells were substantially more abundant. Additionally, compared to cells without co-culture, Jag2OE cells produced larger GFP+ colonies (Fig. 6F). Furthermore, MRK-003 treatment inhibited Jag2OE tumor cells’ growth advantage in the mesothelial co-culture system without affecting the proliferation ability when cultured alone (Fig. 6G-I, Table S3). These findings indicate that Jag2OE tumor cells have an advantage in proliferating when the Notch pathway is activated in mesothelial cells.

### Ectopic expression of Jagged2 enhances chemoresistance in ovarian cancer cells by enhancing cancer stem cell self-renewal (CSCs)

**Fig. 7:**
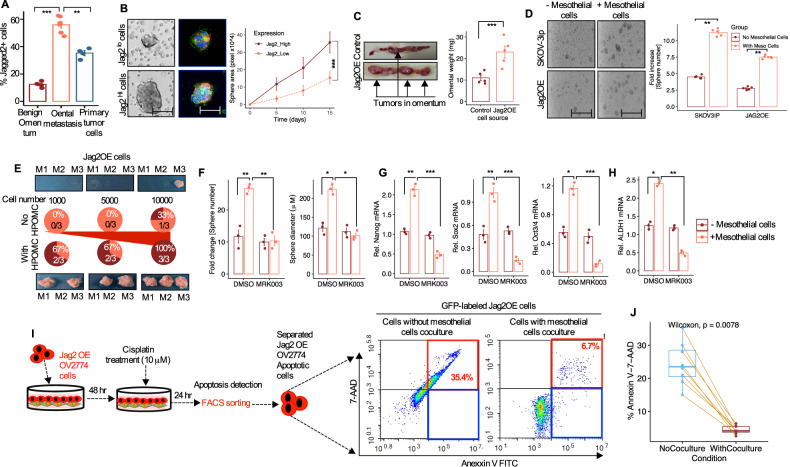
Ectopic expression of Jagged2 in OvCa cells promotes cancer stem cell (CSCs) features and resistance to chemotherapy. **A** Jagged2 expression was examined by fluorescent-activated cell sorting (FACS) in tissues from omentum (*n* = 4) sampled from patients treated for benign disease, OvCa primary tumors (*n* = 4), and omental metastasis (*n* = 4) collected from patients with serous OvCa. Metastatic tumors in the omentum (*n* = 4) had a higher percentage of Jagged2+ cells compared with patients who were either primary tumors or benign omentum (*n* = 4). **P < 0.01, ***P < 0.001 unpaired two-sided *t*-test. **B** Spheroids formation assay comparing cultured omental metastatic (Jag2High) and non-metastatic omentun (Jag2Low) cells. Cells were seeded in ultra-low adherence 96-well plates, and the formation of spheroids was assessed with a wide-filed microscope. (Mean SEM + /-). Scale bar 200 μm. Jagged2 protein immunofluorescently stained with Jagged2 specific primary antibody in cultured spheroids. (Mean SEM + /-). ***P < 0.001, unpaired two-sided *t*-test. **C** (Left)Representative images of tumor localization in mouse omentaum at day 10 after injection of control and Jag2OE cells. (Right) Analysis of tumor burden in omentum at day 10 after injection. Data are presented as mean +/-SD (*n* = 3) and a statistically significant difference was calculated using the Man-Whitney *U* test, ***P < 0.001. **D** Sphere-formation assay of SKOV3-ip and Jag2OE cells after mesothelial cell exposure. Representative sphere images and quantification of sphere numbers fold increase is shown (*n* = 3). Scale bar 200 μm. A statistically significant difference was calculated using the Man-Whitney *U* test, ***P < 0.001. **E** In vivo limiting dilution assay showing tumor formation rate of Jag2OE cells co-injected with mesothelial cells (*n* = 3 mouse/group). **F** Number of spheres and sphere diameter (*n* = 3) in the indicated treatment group. **P < 0.01, ***P < 0.001. **G** qRT-PCR analysis showing relative mRNA expression of the stemness markers, Sox2, Nanog, Oct3/4 in the indicated treatment group. One-way ANOVA test. *P < 0.05, **P < 0.01, ***P < 0.001. **H** ALDH1L1 mRNA in Jag2OE cells after coculture with mesothelial cells and in the indicated treatment groups as normalized to GAPDH mRNA (*n* = 3). *P < 0.05, **P < 0.01. **I** (right) Schematic presentation of the drug response assay designed to assess the proportion of apoptotic cells among the co-cultured OvCa and mesothelial cells. (left) GFP-labeled Jag2OE cells isolated from mesothelial cells were stained with Annexin-V and 7-AAD and the proportion of Annexin-V positive cells was determined by flow cytometry. **J** The percentage of Annexin-V-7AAD positive cells were presented from paired samples (*n* = 8, *P* = 0.0078). *p*-values were analyzed based on Wilcoxon test. Data in the figure represent average and +/-SEM; *P*-values were determined using the Student’s *t*-test unless otherwise indicated.

Ovarian cancer patients have a particularly poor prognosis because of tumor development and metastatic tropism in the omentum. Interestingly, omentectomy prevented the development of the tumor in the omentum in mice, recognizing that the omentum is an important component of the premetastatic niche where tumor cells lay dormant and grow slowly over time (Fig. 2C). Moreover, dormant cells have a long latency for omental tumor development and are also renowned for being resistant to chemotherapy. Tumor-derived Jagged2-mediated omental metastasis, therefore, has two potential pathways that might explain the severity of metastasis and chemotherapeutic resistance of omental malignancies. First, CSC phenotypes within the tumors may be directly influenced by Jagged2-expressing tumor cells. As an alternative, to promote CSC features, Jag2OE tumor cells may synchronize with omental mesothelial cells. Next, Jagged2 expression was assessed using fluorescence-activated cell sorting (FACS) in 4 primary ovarian tumors, 4 omental metastatic HGSC patients’ specimens, and mesothelial cells (isolated from the metastasis-free omentum) in order to test the latter theory and possible enrichment of CSCs. When compared to primary tumor cells and metastasis-free omental mesothelial cells, all omental metastatic samples showed a higher proportion of the Jagged2 positive population (Fig. 7A). Moreover, many CSC markers were overexpressed in omental metastatic OvCa cells compared to cells from primary ovarian tumors (Fig. S4A). Furthermore, an in vitro spheroid assay revealed that Jagged2 high cells (omental metastatic cells) have greater sphere-forming potential than Jagged2 low cells (normal non-metastatic omental cells) counterparts (Fig. 7B). To further demonstrate the role of CSCs in inducing and growing omental metastasis, we implanted Jag2OE cells in a cohort of mice and tracked the tumor burden and metastatic dynamics in the omentum. When compared to control cells, mice bearing Jag2OE tumor cells had a larger total omental tumor burden and a higher number of omental metastatic tumor foci (Fig. 7C; Fig. S4B). Furthermore, Jag2OE cells were associated with the rapid formation of spheroids and an increase in CD44 and ALDH1L1 expression in Jag2OE cells (Fig. S4C). The upregulation of these two critical ovarian stem-cell markers CD44 and ALDH1L1 in omental metastatic tumors points to a potential interaction between the omental mesothelial cell niche and tumor-derived Notch-Jagged2 signaling in the development of CSC features by tumor cells.

To determine whether omental mesothelial cells have any influence on OvCa cell’s stemness and self-renewal, we compared the sphere-forming incidence of OvCa cells cultured alone or together with mesothelial cells. Interestingly, mesothelial cells enhanced the sphere formation ability in Jag2OE and SKOV3-ip cells (Fig. 7D). We next used a gold standard assay to assess the CSC enrichment and self-renewal of the in vitro limiting dilution assay, which mimics the tumorigenic rate with serial dilutions of cancer cells [36]. When OvCa cells were co-cultured with mesothelial cells, sphere formation, and self-renewal behavior were higher at low cell densities than when OvCa cells were cultured alone (Fig. S4D). Similarly, the rate of tumor incidence was investigated in vivo. Tumor incidence was found to be higher at comparably low cell numbers in Jag2OE cells co-injected with mesothelial cells than in OvCa cells alone (Fig. 7E). These findings imply that mesothelial cells enhance the stemness and self-renewal potential of OvCa cells. To illustrate these phenomena at the molecular level, we have shown that mesothelial cells co-cultured with tumor cells significantly enhanced the well-known and well-defined stemness markers Nanog, Oct3/4, Sox2, and ALDH1L1 in OvCa cells (Fig. S4E). Furthermore, mesothelial cells augmented the ALDH1L1 activity in OvCa cells (Fig. S4F). The phenotypes of sphere-forming capability and expression of stemness markers were virtually eliminated when Jag2OE cells were treated with MRK-003 (Fig. 7F-H). This shows that Jagged2, which is produced by tumor cells, promotes CSC self-renewal and, over time, accelerates the development of omental metastatic tumors.

CSCs and dormant cells are known to be resistant to the majority of chemotherapeutic treatments. Considering this, we investigated the platinum sensitivity of OvCa cells grown alone or in indirect co-culture with mesothelial cells to see if omental mesothelial cells influence OvCa cell drug resistance. GFP-labeled OvCa (Jag2OE) cells were cocultured with HPOMCs and treated with cisplatin (Fig. 7I). Co-culture of mesothelial cells reduced cisplatin-induced cell death in OvCa cells in vitro (Fig. 7I). When OvCa cells are co-cultured with mesothelial cells, the number of apoptotic cells is significantly lower than when they are cultured alone under the influence of cisplatin (10 μM) (Fig. 7I). These findings indicate that omental mesothelial cells reduced OvCa cells’ platinum sensitivity. Furthermore, we investigated the cisplatin resistance of Jag2OE cells. We have found that Jag2OE cells were more resistant than control cells (*P* = 0.003) (Fig. S4G). Thus, cells expressing a high proportion of Jagged2 were twice more resistant than control cells (Fig. 7J). Our findings suggest that the Notch pathway, particularly Jagged2, is critical for CSC maintenance and chemoresistance in ovarian cancer omental metastasis.

### Interleukin-6 (IL-6) is secreted by omental mesothelial cells in a Notch and Hes1-dependent manner and stimulates the growth of Jagged2-expressing OvCa cells

**Fig. 8:**
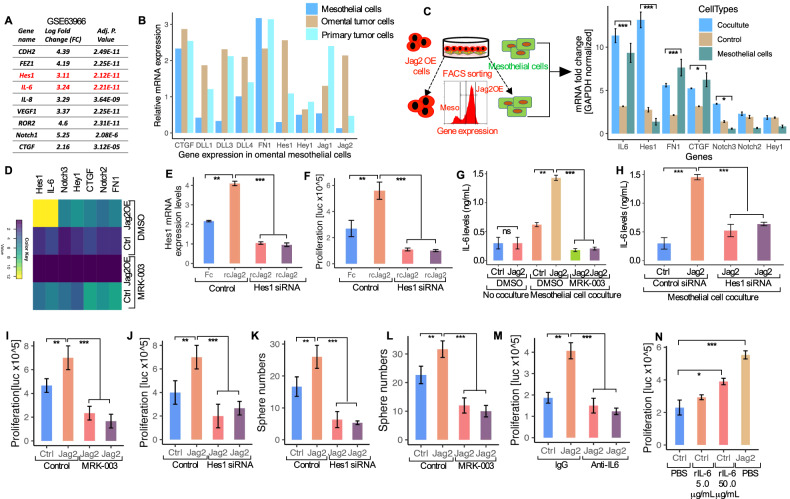
IL-6 secreted from omental mesothelial cells induce stemness in a Notch-Hes1 dependent fashion and stimulates the growth of Jagged2 expressing cells. **A** A List of genes with an expression fold change between pleural malignant and peritoneal malignant mesothelial cells of more than 2-fold from the public dataset microarray GSE63966. **B** qRT-PCR mRNA analysis of the Notch target genes, and CTGF and FN1 from mesothelial cells, omental metastatic tumor cells, and primary ovary tumor cells resected from the patient. **C** (Left) Schematic model of the co-culture system of mesothelial and Jag2OE cells. (Right) qRT-PCR mRNA expression of the indicated genes in control and Jag2OE tumor cells cocultured with mesothelial cells. *P < 0.05, **P < 0.01, ***P < 0.001. **D** Heat map showing qRT-PCR mRNA expression levels of the indicated genes from mesothelial cells that were FACS separated from cocultures of each experimental/treatment group. **E** qRT-PCR analysis of Hes1 expression in mesothelial cells treated with scrambled or Hes1 siRNA and cultured in 24 well plates coated with either Fc or control or recombinant Jagged2 protein (*n* = 3). Data represent average +/- SD. **P < 0.01, ***P < 0.001. **F** Quantification of cell proliferation in mesothelial cells treated with control or Hes1 siRNA and cultured in 24 well plates coated with either Fc or control or recombinant Jagged2 protein by luciferase assay (*n* = 3). **P < 0.01, ***P < 0.001. **G** Quantification of IL-6 levels in the conditioned media of control or Jag2OE tumor cells cultured alone or cocultured with mesothelial cells in the presence of DMSO, MRK-003 (1μM) by ELISA (*n* = 3). **P < 0.01, ****P < 0.001. **H** Quantification of IL-6 levels in the conditioned media of the indicated tumor cells co-cultured with mesothelial cells after treatment with Hes1 siRNA by ELISA. ***P < 0.001. **I** Cell proliferation of the indicated tumor cells co-cultured with mesothelial cells from each experimental group by luciferase assay. Data represent average +/-SD, **P < 0.01, ***P < 0.001. **J** Cell proliferation of the indicated tumor cells cocultured with mesothelial cells after treatment with Hes1 siRNA by luciferase assay. **P < 0.01, ***P < 0.001. **K** Quantification of sphere formation capacity of the indicated tumor cells cocultured with mesothelial cells after treatment with Hes1 siRNA. **P < 0.01, ***P < 0.001. **L** Quantification of sphere formation capacity of indicated tumor cells cocultured with mesothelial cells after treatment with 1 μM MRK-003. **P < 0.01, ***P < 0.001. **M** Cell proliferation of indicated tumor cells cocultured with mesothelial cells and treatment with either IgG or 5.0 μg/mL of the anti-IL-6 antibody by luciferase assay. **P < 0.01, ***P < 0.001. **N** Cell proliferation of indicated tumor cells cocultured with mesothelial cells and treatment with either PBS, 5.0 μg/mL, or 10.0 μg/mL of anti-IL6 by luciferase assay. *P < 0.05, ***P < 0.001. Data in the figure represent average and +/-SEM; *P*-values were determined using the Student’s *t*-test unless otherwise indicated. All experiments were run in triplicates.

The predominant cell type in direct contact with OvCa cells in the peritoneal cavity is the omental mesothelial cells [12]. Jagged2-regulated genes in mesothelial cells may create a microenvironment favorable for tumor development and survival. To determine the dynamics of these tumor cells during omental metastasis, we first examined several promising Notch-dependent candidate genes expressed by omental mesothelial cells from a previously reported dataset (GSE63966). The Dragon dataset [37] revealed that IL-6 is the most promising candidate gene from the ranked-in-order gene list (Fig. 8A), owing to its involvement in chemoresistance, omental metastasis [38, 39], and its association with poor clinical outcome in OvCa [40]. Therefore, the expression of the Notch ligands was assessed in mesothelial cells obtained from benign omental mesothelial cells, primary ovarian tumors, and cells purified from patients’ omental metastatic tumor tissues in order to identify Notch-dependent signaling molecules secreted by mesothelial cells and tumor cell that can stimulate omental tumor growth. Figure 8B shows that multiple genes are overexpressed in these cells (*P* < 0.01, Mann-Whitney test). We have used qRT-PCR on FACS-sorted cells that were co-cultured to uncover Jagged2-regulated genes in mesothelial cells that are particularly required for omental tumor growth (Fig. 8C). Multiple genes, including IL-6 and several Notch target genes, are activated in mesothelial cells co-cultured with Jag2OE cells compared to the control group (Fig. 8C). Interestingly, when these cells were exposed to MRK-003, all of these genes were attenuated (Fig. 8D). We next examined Hes1, which is well known as a downstream mediator of the Notch pathway, which also plays important roles in stemness, metastasis, and multi-drug resistance; and has been found highly expressed in omental mesothelial cells [41–43]. We have inhibited Hes1 expression in mesothelial cells to verify its requirements (Fig. 8E). The growth of Jag2OE tumor cells in co-culture was markedly diminished by Hes1 knockdown in mesothelial cells (Fig. 8F). This shows that the downstream Notch pathway mediator Hes1 is essential for the development of omental metastatic tumors.

The growth and survival of OvCa cells, as well as their ability to adhere and metastasize, all depend on intimate interactions with their microenvironment. We then investigated the Notch-dependent signaling molecule released by omental mesothelial cells, which may boost tumor growth, in light of the reciprocal contact between tumor and omental mesothelial cells. The transcriptome profile of HGSOC tumors from the TCGA data portal revealed that an increase in IL-6 expression was strongly related to poor tumor-free survival and played a key role in CSC self-renewal and maintenance, treatment resistance, and tumor invasion [44–46]. Furthermore, IL-6 was identified as a promising candidate gene in selective gene expression analysis from publicly available datasets (Fig. 8A). IL-6 levels increased 3-4-fold when Jag2OE cells were co-cultured with mesothelial cells (Fig. 8G). Importantly, conditioned media from tumor cells cultured alone produced low amounts of IL-6, and IL-6 secretion from mesothelial cells was dependent on the Notch signaling, as demonstrated by the MRK-003 treatment (Fig. 8G). This implies that the ability of tumor development in the metastatic site by Jag2OE cells is exclusively dependent on the existence of mesothelial cells, which act as a reservoir for inflammatory stimuli such as IL-6. We then established that Hes1 regulates IL-6 expression (Fig. 8H). Importantly, MRK-003 and Hes1siRNA treatment inhibited Jag2OE tumor cell proliferation and self-renewal ability in mesothelial co-culture (Fig. 8I–L). Given the relationship between the Notch pathway proteins and IL-6 and based on our findings stated above, we have investigated whether Notch-activated IL-6 secretion from omental mesothelial cells was necessary to promote tumor proliferation, stemness, and self-renewal features. To investigate this, we used a neutralizing antibody to suppress mesothelial cell-derived IL-6 release. The reduction of IL-6 production by mesothelial cells reduced the growth advantage of Jag2OE tumor cells (Fig. 8M). Furthermore, adding rIL-6 to control tumor cells greatly increased their proliferation capacity (Fig. 8N). Collectively, these findings show a positive feedback loop in which the Jagged2-Notch signaling boosts IL-6 production by mesothelial cells, promoting the formation of metastatic tumors in the omentum.

The tumor-associated stroma, which includes mesothelial cells, fibroblasts, and adipocytes, serves as a reservoir for IL-6 and contributes to the establishment of favorable niches for tumor cell growth, and chemotherapeutic resistance, and the maintenance of OCSCs (ovarian cancer stem cells). To investigate the role of the microenvironment in cisplatin-induced IL-6 secretion, mesothelial cells were grown alone or co-cultured with Jag2OE cells under starvation conditions for 24 hours before being treated with cisplatin (10 μM). IL-6 levels in the conditioned media were measured 24 hours after exposure to cisplatin. Cisplatin treatment enhanced IL-6 production in mesothelial cells relative to untreated cells, indicating a functional role of IL-6 released by mesothelial cells in the resistance to cisplatin (Fig. S5A). Furthermore, co-culturing Jag2OE and SKOV3-ip cells with mesothelial cells enhanced IL-6 secretion (Fig. S5A), indicating a role for mesothelial cells in cisplatin-induced IL-6 secretion. To test whether IL-6 is required and sufficient to enhance mesothelial cell-mediated OvCa cell chemoresistance and self-renewal traits, we treated Jag2OE cells with rIL-6 and examined the chemoresistance and self-renewal phenotypes. Exogenous rIL-6 treatment boosted Jag2OE cells’ cisplatin resistance as well as their capacity to develop spheres (Figs. S5B, S5C). The suppression of IL-6 from mesothelial cells reduced the sphere growth advantage of Jag2OE tumor cells (Fig. S5D). These findings point to IL-6 as a crucial component in OvCa chemoresistance and elevated stemness features mediated by mesothelial cells.

### Disruption of the Notch signaling pathway by the Notch inhibitor MRK-003 impairs the omental metastatic spread of ovarian cancer

**Fig. 9:**
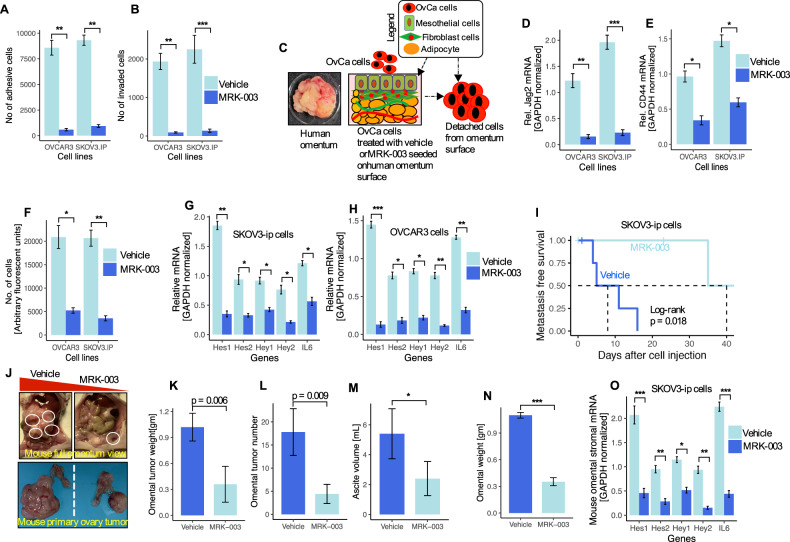
Disruption of the Notch signaling pathway with MRK-003 prevents the omental metastatic spread of ovarian cancer. **A**, **B** OvCa cells were treated with vehicle and MRK-003 (5 μM), followed by the addition of fluorescently labeled SKOV3-ip and OVCAR3 cells and later detected by fluorescent reader. **P < 0.01, ***P < 0.001. **C** The experimental model of vehicle or MRK-003 (5 μM) treated and fluorescently labeled OvCa cells seeded in a piece of human omentum (72 hours). **D–E** Jagged2 inhibition by MRK-003 as described in (**A** and **B**). After scraping off the omentum surface cells of the omentum, Jagged2 (**D**) and CD44 (**E**) were quantified using qRT-PCR. *P < 0.05, ***P < 0.001. **F** Quantification of the number of cells after scrapping from the omentum. *P < 0.05, **P < 0.01. **G**-**H** qRT-PCR mRNA analysis of the Notch target genes and IL-6 from scraped-off cells from the omentum. *P < 0.05, **P < 0.01, ***P < 0.001. **I** Kaplan-Meier omental metastasis-free survival curve of mice from each experimental group. (Log-rank *P* = 0.018). **J** Representative images of tumor metastasis to omentum (top; highlighted with white circles) and primary ovary site (bottom) in female mice injected with SKOV3-ip cells and treated with vehicle or MRK-003. **K–N** Omental tumor weight (**K**), omental tumor number (**L**), ascites volume (**M**), and omental weight (**N**) at the endpoint of mice intraperitoneal inoculation. *P* = 0.006 and 0.009; *P < 0.05, ***P < 0.001. **O** qRT-PCR analysis of the Notch target genes and IL-6 in the tumor-stromal compartment of omental metastasis from vehicle or MRK-003 treated mice. *P < 0.05, **P < 0.01, ***P < 0.001. Data in the figure represent average and +/-SEM; *P* values were determined using the Student’s *t*-test unless otherwise indicated. All experiments were run in triplicates.

The experimental results have thus far drawn attention to and pinpointed the function of the Notch signaling system in promoting an omental metastatic niche that is supported by contact between the tumor and mesothelial cells. As a result, the therapeutic intervention of Notch signaling may compromise tumor-mesothelial cell integrity, impairing metastatic implantation in the peritoneum, omentum, and beyond, and may be a promising strategy for the suppression or decrease of metastatic tumor development. To determine the Notch inhibitor MRK-003’s ability to reduce omental metastasis, we primarily focused on the omental microenvironment that promotes metastasis. We tested this using two methods. First, we treated OvCa cells with MRK-003 to prevent the production of Jagged2. We have shown that OvCa cells did not adhere (92% of control) or invade (89% of control) as efficiently (Fig. 9A, B). In a second technique, MKR-003-treated OvCa cells were seeded on freshly resected human omentum. After three days of incubation, the MRK-003-treated OvCa cells were able to attach; nevertheless, cells removed from the omentum revealed lower expression of the Jagged2 mRNA and CD44 (Fig. 9C-F). Furthermore, MRK-003 treatment decreased the expression of the Notch target genes, including IL-6 (Fig. 9G, H). This indicates the necessity for cross-talk between OvCa cell-derived Jagged2 and the supporting omental microenvironment and mesothelial cells.

To confirm these results in vivo, female nude mice were intraperitoneally injected with metastatic SKOV3-ip cells, which express high levels of endogenous Jagged2 (Fig. 3G), and then treated with MRK-003. Interestingly, MRK-003 treatment delayed the onset of omental metastases significantly (Fig. 9I; log-rank *P* = 0.018). The vehicle treatment did not affect the influx of OvCa cells into the omentum while greatly reduced peritoneal and omental tumor nodules (Fig. 9J), omental tumor weight and numbers (Fig. 9K, L), ascites volume (Fig. 9M), and overall omental weight (Fig. 9N). To our surprise, MRK-003 treatment did not affect the development of primary tumors in mouse ovaries (Fig. 9J), indicating that direct inhibition of the Notch signaling in tumor cells might not be very effective in preventing the development of primary tumors. This would explain the necessity of the omental microenvironment in omental metastasis. We have next evaluated the expression of the Notch target genes and IL-6 to determine if MRK-003 treatment impairs the Notch signaling in the omental stromal compartment. As measured by decreased expression of the Notch target genes in the omental stromal compartment, MRK-003 treatment significantly disrupted the Notch signaling (Fig. 9O). Collectively, these findings indicate that targeting Jagged2, a tumor-derived protein, reduces colonization and metastasis in the omentum and beyond.

### MRK-003 reverses the omental metastatic phenotype induced by Jag2OE cells

**Fig. 10:**
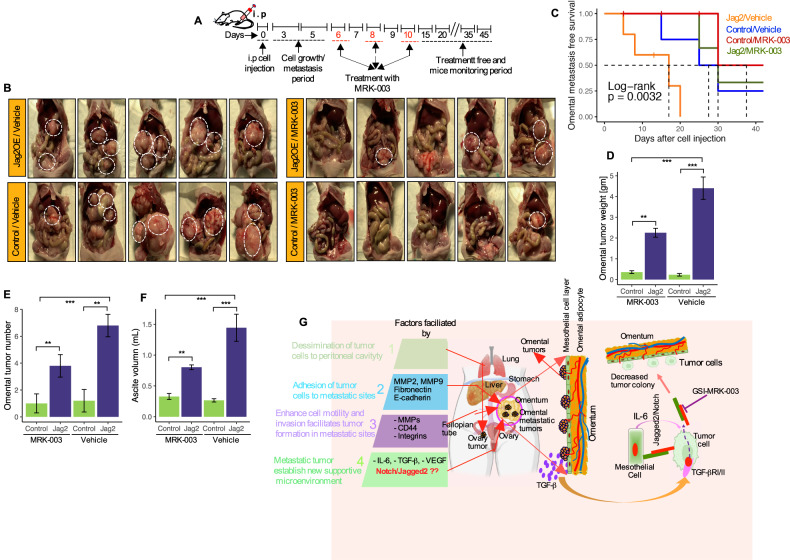
Inhibiting the Notch signaling pathway with MRK-003 reverses Jagged2-mediated omental metastasis. **A** Schematic diagram of i.p. cell injection and treatment schedules. **B** Images of representative mice in each experimental group on day 40 after peritoneal injection. **C** Kaplan-Meier omental metastasis-free survival curve of mice from each experimental group. Log-rank *P* = 0.0032. **D–F** Omental tumor weight (**D**), omental tumor number (**E**), and ascites volume (**F**) of mice from each experimental group (mean +/- SEM, n = 5 for each experiment, **P < 0.01, ***P < 0.001, Students *t*- test). **G** A graphical presentation of tumor-mesothelial cell cross-talk of Jagged2-expressing ovarian tumor cells with the omental microenvironment. Data in the figures represent average and +/-SEM; *P*-values were determined using the Student’s *t*-test unless otherwise indicated. All experiments were run in triplicates.

After confirming the influence of Jagged2 on the omental metastasis, we tested the possible role of MRK-003 treatment in reversing the omental metastatic phenotypes, which were driven by Jag2OE cells. Accordingly, we have first investigated the effect of MRK-003 on the expression of the Notch target genes. In fact, the levels of these genes were found to be higher in the Jag2OE cells (Figs. S6A–SC). However, following MRK-003 treatment, the expression of the Notch target genes was markedly reduced. Likewise, MRK-003 has inhibited cell invasion and sphere formation ability (Figs. S6B–SC). In line with these alterations, the omental metastasis was also diminished in MRK-003-treated animals by dramatically reducing the metastatic time, tumor mass, number of omental tumors, and ascites volume (Fig. 10A–F). These findings underscore the contribution of tumor-derived Jagged2 to the dissemination and omental metastases of OvCa. The results taken together indicate that the omental metastases mediated by Jagged2-expressing ovarian cancer cells are dependent on coordinated mesothelial-stromal Notch activation and that this pathway may be pharmacologically inhibited to prevent these metastases from occurring in the omental milieu.

## Discussion

Metastatic ovarian cancer is a predominant cause of ovarian cancer-related fatalities. Emerging evidence suggests that the cross-communications between the tumor-omental microenvironment (TME) is crucial for the development of both metastatic tumor and therapeutic resistance [47, 48]. The cellular dynamics that explain how the omentum and omental mesothelial cells contribute to the establishment of a pre-metastatic niche and the cross-talk between the omental mesothelial and cancer cells have only been sparsely studied. In this study, we have demonstrated that elevated Jagged2 expression in ovarian tumor cells promotes omental metastasis by activating the Notch signaling pathway, which is dependent at least in part on omental mesothelial cells. Jagged2 is overexpressed in ovarian metastatic tumor cells, which are further activated by TGF-β from mesothelial cells during the onset of metastatic tumor growth. Jagged2-expressing ovarian cancer cells interact with mesothelial cells and acquire a growth advantage in the tumor microenvironment by stimulating and releasing of IL-6 from omental mesothelial cells. Conversely, GSI (γ-secretase inhibitor) MRK-003 treatment, reversed these premetastatic functions of Jagged2 by impairing the Notch signaling pathway in associated omental mesothelial cells. Our findings identified omental mesothelial cells as a critical component of the ovarian TME, which supports metastatic tumor development and provides a unique paradigm for the active participation of the Notch signaling pathway in the growth and progression of omental metastatic tumors (Fig. 10G).

Notch, a conserved signaling pathway, has been implicated in the maintenance of tissue homeostasis by regulating self-renewal and cell fate determination in normal stem cells and early progenitor cells. Many studies, including those using TCGA data, have found that the Notch pathway is dysregulated in OvCa [49]. A role for the Notch signaling in ovarian cancer was originally reported in two separate studies that investigated the expression of the Notch receptors and ligands [50, 51]. The majority of the subsequent studies on the implication of Notch signaling in ovarian cancer progression focused on its activation in tumor cells and the accompanying interaction between the Notch receptors and downstream targets in the context of cellular proliferation, differentiation, and apoptosis. However, it is important to note that the Notch signaling is aberrantly active in many solid tumors, raising the possibility that the Notch receptors are activated upstream through ligand interaction, implicating both signal-sending and signal-receiving cells [52]. The growth and survival of tumor cells, as well as their propensity to metastasize, are dependent on complex interactions with their distinct microenvironments.

Despite prior studies indicating that DLL4 regulates tumor dormancy [52] while, mesothelial cell-derived Jagged2 forms a juxtracrine loop with the Notch receptor expressed by OvCa cells and regulates tumor growth and adhesion [12]. The precise mechanism and cellular dynamics that create the metastatic niche, especially in the omentum, that describe the involvement of the Notch pathway ligands, particularly Jagged2, and ovarian cancer metastasis in the omentum are mostly unknown despite the existence of these reports. Recognizing the significance of the tumor-associated milieu, we explored the possibility that omental mesothelial cells play an active role in the metastatic cascade. Our findings made the exciting revelation that ovarian cancer metastasis is not largely caused by the Notch pathway receptors and its downstream targets. Additionally, we have demonstrated for the first time that high levels of OvCa cells’ capacity for omental metastasis correlate with elevated levels of the Notch ligand Jagged2, which is produced by the tumor cells and is likewise associated with a poor prognosis.

Growing evidence indicates that the Notch pathway not only plays a crucial role in a myriad of developmental processes, but it’s also implicated in the tumor-associated stroma, which facilitates cancer progression [41]. There is evidence that mesothelial cells play an active role in the establishment of the ovarian cancer niche, and cancer cells recruit local stromal cells to promote and sustain tumor cell growth [3, 53]. According to a recent study, inflammatory factors secreted by OvCa cells mobilize neutrophils and stimulate them to create neutrophil extracellular traps (NETs) in the omentum in both tumor-bearing mice and early-stage OvCa patients, and the NETs then capture OvCa cells, promoting metastasis formation [54]. Furthermore, Etzerodt et al. (2020) demonstrated that tissue-resident macrophages promote the metastatic progression of OvCa in the omentum. But, the significance of Notch signalling in the tumor-associated stromal microenvironment is largely unexplored. In the present study, we found that Jagged2 was not detected in the tumor-free omentum but was found in the omentum of women with ovarian tumors. These findings support the notion that the interaction between tumor and mesothelial cells in the omentum is reflective of the metastatic potential, which is initiated by tumor-derived Jagged2. Furthermore, we present here the first indication that tumor-derived Jagged2 can facilitate the growth of omental tumors by activating the Notch signaling pathway in omental mesothelial cells. This, in turn, promotes tumor cell adherence and growth advantage in the omental milieu in both mouse models and OvCa patients. These findings implicate that mesothelial cells activate the Notch pathway in the pre-metastatic niche in response to the presence of OvCa cells. While we did not assess the role and involvement of additional omental and stromal cell types, such as adipocytes, fibronectin, and endothelial cells in Jagged2-mediated omental metastasis, our study nevertheless warrants further investigative work into these cells’ potential roles.

Despite initial chemosensitivity of HGSOCs, relapse of the disease precludes cures and metastasis in the majority of patients. This may be attributed, at least in part, to the cancer stem cells (CSCs) acquiring stem-like features, which have been linked to promoting tumor progression and metastasis [55]. The growing body of evidence supports the role of CSCs in intraperitoneal metastasis, chemo- and radio-resistance, which has major implications for disease recurrence from disseminated tumor cells [23, 48, 56, 57]. Likewise, CSCs have anchorage-dependent survival and are dynamically altered by signal queues from the tumor microenvironment [58]. Several studies have confirmed that CSCs are present in ascitic tumor cells in advanced-stage ovarian cancer [56]. It has recently been found that the Notch signaling molecules play a significant role in CSC maintenance in a variety of malignancies, including ovarian cancer [24, 59]. It has been demonstrated by several recent investigations that Jagged1 is essential for preserving the stem cell phenotypes in the stroma. Other Notch ligands, such as Jagged2 and their functions and involvement in stem cell maintenance, have not yet been explored. Interestingly, our investigation of the patient’s omental metastatic tumor sample analysis revealed a modest increase in the expression of CSC markers. Our co-culture investigations, on the other hand, indicated that Jagged2 induces the production and secretion of IL-6 from mesothelial cells *via* activation of the Notch signaling pathway, imparting mesothelial cell-dependent CSC growth and proliferation advantage to omental metastatic tumor cells. Recent studies have shown that the IL-6/STAT3 signalling promotes CSC self-renewal and maintenance in ovarian cancer cells and supports tumor growth in the omental microenvironment [38, 48]. Several lines of evidence documented that IL-6 levels in the serum and peritoneal fluids are higher in ovarian cancer patients, and high level of IL-6 is independently associated with poor prognosis and survival of these patients [60, 61]. We have shown here that mesothelial cells produce IL-6 to enhance ovarian CSCs in the omental milieu and promote omental metastasis. Importantly, we have observed that metastatic omental tumors increased CSC marker expression and activity, implying that IL-6 actively functions in the omental microenvironment. Our findings further show that the mesothelial cell-dependent positive feedback mechanism of IL-6 further extends its participation in Jagged2-mediated omental metastasis. Future research is required to fully understand how mesothelial cells in the omental microenvironment promote CSC expression in ovarian cancer cells, as well as the molecular mechanisms of the dialogue between tumor-derived Jagged2 and dormant ovarian cancer stem cells’ self-renewal during the metastatic events.

The importance of cancer cell-derived TGF-β during OvCa omental metastasis has been extensively studied. The omental mesothelial cells can support cell adhesion, proliferation, invasion, and migration through their rich reservoir of TGF-β, which can be released into the omental microenvironment during aggressive omental metastasis. TGF-β has been found to interact with peritoneal mesothelial cells and activate the RAC/SMAD signaling pathway, resulting in enhanced fibronectin production and a mesenchymal phenotype of peritoneal mesothelial cells [3]. The disruption of the TGF-β signalling, either pharmacologically or genetically, may reduce the formation of ovarian cancer metastases on the omentum, highlighting the role of the TGF-β signaling system in supporting tumor cells’ omental metastatic capacity [35, 62, 63]. The functional downstream targets of the TGF-β-SMAD pathway in the metastasis of ovarian cancer are not well understood. We are the first have shown in this study that Jagged2 is a SMAD-dependent target of TGF-β in omental metastasis, given the particular involvement of TGF-β in omental metastasis. Thus, it is likely that in response to mesothelial cell-derived TGF-β signaling during metastasis, Jagged2 triggers a positive feedback loop, which may promote the activation of the Notch signaling in TME, once the TGF-β1 is upregulated during metastasis. Furthermore, the administration of a neutralizing antibody effectively restricts the feedback of TGF-β on Jag2OE tumor cell and mesothelial cell co-cultures without altering the growth properties of cells. Our findings highlight an important fact that the release of TGF-β from mesothelial cells is pivotal in the metastasis process in response to the activation of the Notch signaling pathway, and it is likely to have an important role in the pathogenesis of Jagged2-mediated omental metastasis. It is unknown how the TGF-β-Notch signaling promotes metastasis. However, the role of the components of this pathway in the context of epithelial-to-mesenchymal transition has been documented [64]. Our findings reveal that the activation of both Notch and TGF-β signaling pathways built a positive feedback bridge between tumor cells and the omental milieu, promoting omental metastasis.

Pharmacological inhibitors of γ-secretase are being developed for clinical use as a single treatment or in combination with chemotherapy in order to inhibit the Notch signaling pathway. This approach is garnering tremendous attention. Targeting γ-secretase prevents NICD from being cleaved and released because γ-secretase causes the proteolytic release of the NICD receptors. GSIs have been found to suppress cancer cell proliferation and tumor progression. However, it has not been determined whether tumor progression is impeded by altering the Notch signaling as well as the related stromal-tumor milieu. We employed multiple methodologies, each with a specific focus on tumor and mesothelial cells, to investigate how disrupting Notch signaling by MRK-003, a GSI inhibitor, impairs Notch-Jagged2-mediated cross-talk between tumor cells and mesothelial cells. The obtained data show that MRK-003 may be an effective strategy for limiting ovarian cancer omental metastasis by inhibiting the Notch signaling. However, GSIs must be used with caution since, similar to other chemotherapeutic drugs, cancer cells have the potential to develop resistance to GSI therapy.

## Conclusions

In summary, although the story of omental metastasis and tumor-stromal cross-talk was initiated by tumor-derived Jagged2, the findings reported here revealed that the real path forward actually lies in a significant stroma-dependent mechanism for the Notch ligand Jagged2 in promoting ovarian cancer metastasis on the peritoneal omentum. Our findings further show how cross-talk between ovarian tumor cells and omental mesothelial cells, which promote ovarian cancer metastasis in the omentum, is bridged by two developmentally different TGF-β-Notch signaling pathways. Additionally, by focusing on the tumor-stroma environment, we have shown preclinical evidence for MRK-003 as a therapeutic drug that effectively prevents omental metastasis. These exciting preclinical findings merit further investigation to gain new insights that can help treat and prevent the spread of ovarian cancer as well as reduce the morbidities associated with metastasis.

### Study approval

All procedures involving animals were performed in accordance with guidelines issued by the institutional animal care and use committees at the King Faisal Specialist Hospital and Research Centre under approved animal use protocols (RAC#2170034). For human studies, the study was approved by the local ethics committee of the King Faisal Specialist Hospital and Research Centre and was conducted in accordance with the declaration of Helsinki. All participants provided written informed consent

## Data and materials availability

All pertinent data required to evaluate the conclusions in the manuscript are present in the paper or the supplementary materials.

### Supplementary information

Supplemental Materials, Figures and Legends

Reproducibility_Check_List_DIS-23-3935-T

Raw western blots
