# Supplementary material for: Ovarian tumor cell-derived JAGGED2 promotes omental metastasis through stimulating the Notch signaling pathway in the mesothelial cells
Source: Cell Death Dis. 2024 Apr 4;15(4):247. doi: 10.1038/s41419-024-06512-0 (PMC10995149; doi:10.1038/s41419-024-06512-0)
Supplement: Supplementary file 1 — Supplemental Materials, Figures and Legends [file 41419_2024_6512_MOESM1_ESM.pdf]

Supplementary materials for

Ovarian tumor cell-derived JAGGED2 promotes omental metastasis through stimulating the Notch signaling pathway in the mesothelial cells

Syed S Islam *et al.*

*Corresponding author: sislam83@kfshrc.edu.sa

This PDF file includes
Supplementary Figures S1 to S6
Supplementary Table S1, S2 and S3

Supplementary Figure S1 of Figure 1: Isolation of human omental mesothelial cells and growth analysis

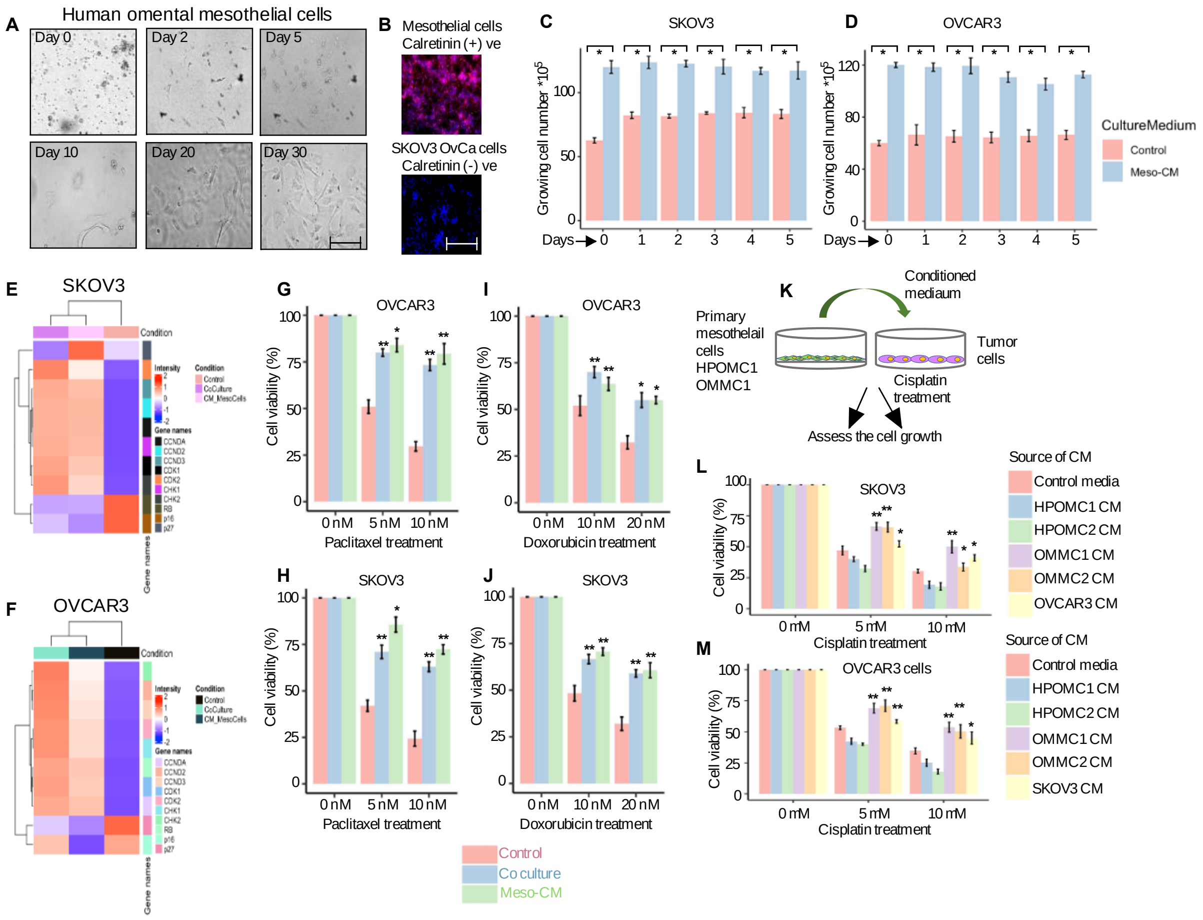

Supplementary Figure S1 of Figure 1. Isolation and culture of mesothelial cells from a human metastatic omentum resected from OvCa patient.

A Phase contrast microscopy of primary human mesothelial cell culture from day 0 to day 30. Scale bar 100 µm (x40 bar in the figure)**.**

B Immunofluorescence assay shows the positive staining of mesothelial cell marker Calretinin and negative staining in SKOV3 tumor cells. Scale bar 100 µm (x40 bar in the figure)**.**

C-D SKOV3 and OVCAR3 cells were co-cultured with mesothelial cells in a co-cultured transwell system for the indicated period and then counted the cell number. $*P < 0.05$.

E-F Real-time RT-PCR analysis of multiple cell cycle regulator gene expression in the indicated cell lines and groups at day 5 of culture. Pseudo-color scale values in the heatmap

graphs were log-transformed. All transcript levels were normalized against GAPDH expression. The experiments were independently performed three times.

G-J Cell viability of SKOV3 and OVCAR3 cells treated with the indicated concentrations of paclitaxel and doxorubicin in the indicated three culture conditions *$P < 0.05$, **$P < 0.01$.

K Schematic model for coculture system

L-M SKOV3 and OVCAR3 cells were cultured in a conditioned medium from normal human primary omental mesothelial cells (HOPMC), or human omental metastatic mesothelial cells (OMMCs) and then were treated with cisplatin for 48 hours and assessed the cell viability as depicted in the experimental design. For statistical quantification, cell viability was normalized against the untreated control (n = 3), *$P < 0.05$, **$P < 0.01$.

Data in the figure represent average and +/-SEM; p- p-values were determined using the Student's *t*-test unless otherwise indicated.

Supplementary Figure S2 of Figure 3: Notch receptors and ligand expression and Relapse free survival from TCGA datasets

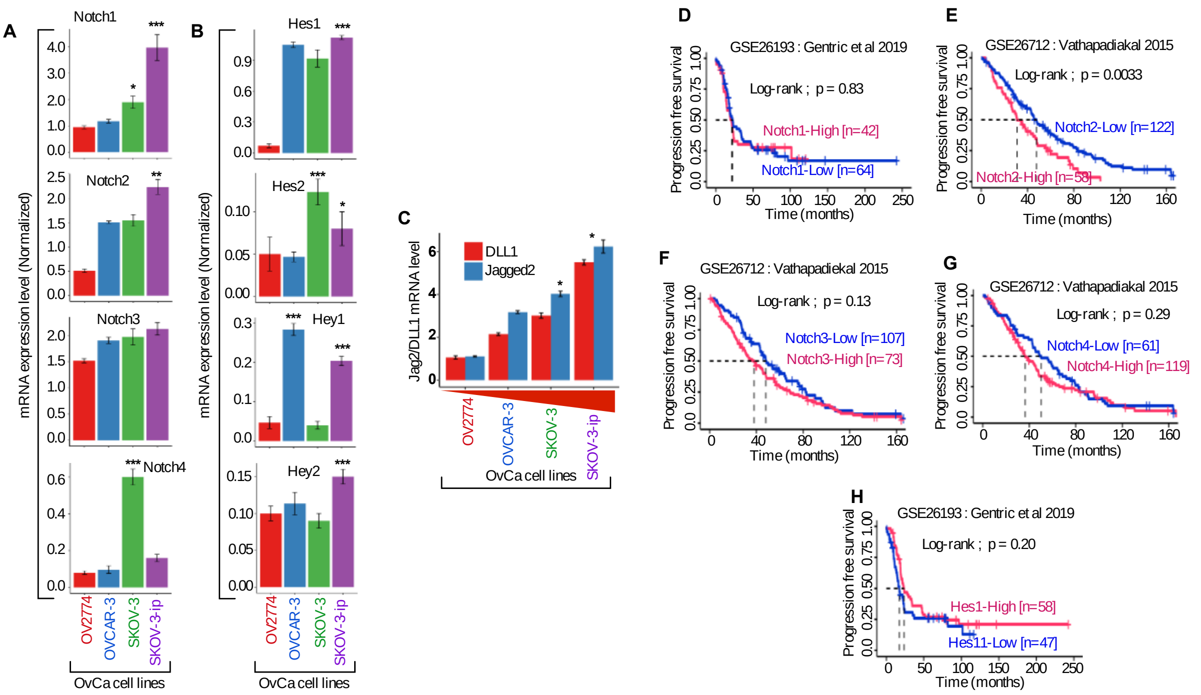

Supplementary Figure S2 of Figure 3.

A qRT-PCR mRNA expression of the Notch ligands Notch1, Notch2, Notch3, and Notch4 in the indicated ovarian cancer cell lines.

B qRT-PCR mRNA expression of the Notch downstream targets Hes1, Hes2, Hey1, and Hey2 in the indicted ovarian cancer cell lines.

C Jagged2 and DLL1 mRNA expression in the indicated ovarian cancer cell lines.

D-H Kaplan-Meier progression-free survival curves of patients from Gentric (GSE26193) (Gentric et al., 2019) and Vathipadiekal (GSE26712) (Vathipadiekal et al., 2015) data sets presented as high and low expression of each gene.

Data in the figure represent average +/- SEM, SD: p-values were analyzed based on the student's t-test. Experiments were run in triplicates for Figures **A-C**.

Supplementary Figure S3 of Figure 5: Jag2 is functionally regulated by TGF-beta-Smad3 signaling pathway in ovarian cancer omental metastasis

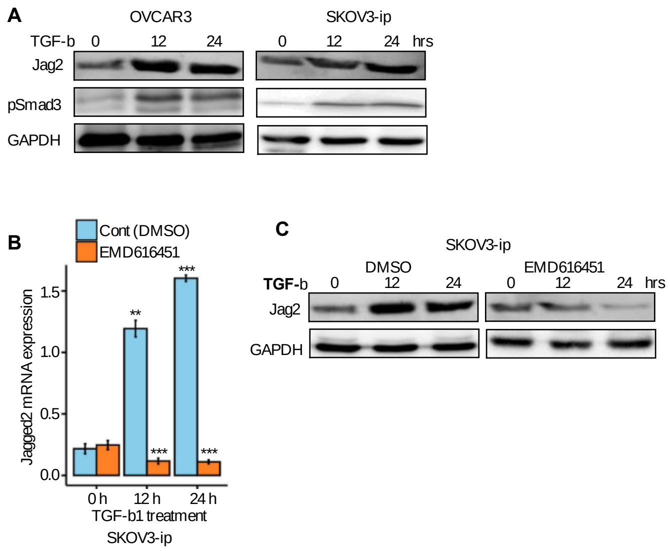

Supplementary Figure S3 of Figure 5

A Western blot analysis of Jagged2 and p-Smad3 in response to TGF-β treatment in the indicated time in OvCa cells. Cells were treated with 6nM of TGF-β as shown, and then whole cell lysates were used for immunoblotting analysis.

B-C Jagged2 mRNA and protein expression in response to indicated time-course of TGF-β treatment with or without TGF-β receptor inhibitor EMD616451.

Data in Figure **(B)** represent average and +/-SEM; p-values were determined using the Student's *t*-test. **i < 0.01, ** **P* < 0.001.

Data in the figure represent average and +/- SEM. *P*-values were determined using the Student's *t*-test unless otherwise indicated.

Supplementary Figure S4 of Figure 7: Enforced expression of Jagged2 in ovarian cancer elevate CSC capability and resistant to chemotherapy

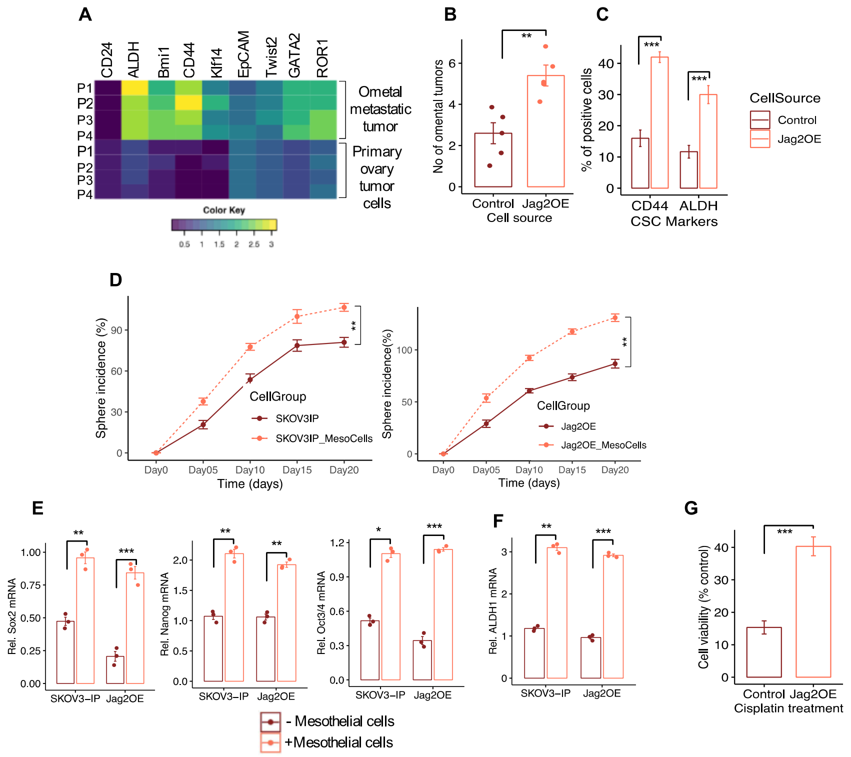

Supplementary Figure S4 of Figure 7. Ectopic expression of Jagged 2 in ovarian cancer cells elevates CSC capability and resistance to chemotherapy

A Heat map depicting gene expression of cancer stem cells (CSCs), epithelial-to-mesenchymal markers in human omental metastatic tumor cells, and tumor cells from the primary ovarian tumors.

B Quantification of the number of tumors in the omentum in each experimental group. Statistical significance difference was calculated using the Man-Whitney *U* test, $^{**}P < 0.01$, $^{**}P < 0.01$.

C Quantification of the percentages of CD44 and ALDH-positive cells in each experimental group. $^{***}P < 0.001$.

D Effects of mesothelial cell co-cultures on SKOV3-ip and Jag2OE cell sphere formation incidence. $^{**}P < 0.01$.

E qRT-PCR analysis showing relative mRNA expression of the stemness markers, Sox2, Nanog, Oct3/4 after co-culture with mesothelial cells. One-way ANOVA test. *$P < 0.05$, **$P < 0.01$, ***$P < 0.001$.

F ALDH1 mRNA in Jag2OE cells after coculture with mesothelial cells as normalized to GAPDH mRNA (n = 3). **$P < 0.01$, ***P < 0.001.

G Viability of Jag2OE and control cells after cisplatin treatment (10 μM) in each experiment. ***i < 0.001. Data in the figure represent average and +/-SEM; *P*-values were determined using the Student's *t*-test unless otherwise indicated. All experiments were run in triplicates.

Supplementary Figure S5 of Figure 8: IL-6 secreted by mesothelial cells induces stemmness and chemoresistance in a Notch-Hes1 dependent fashion and stimulate growth of Jag2 expressing tumor cells

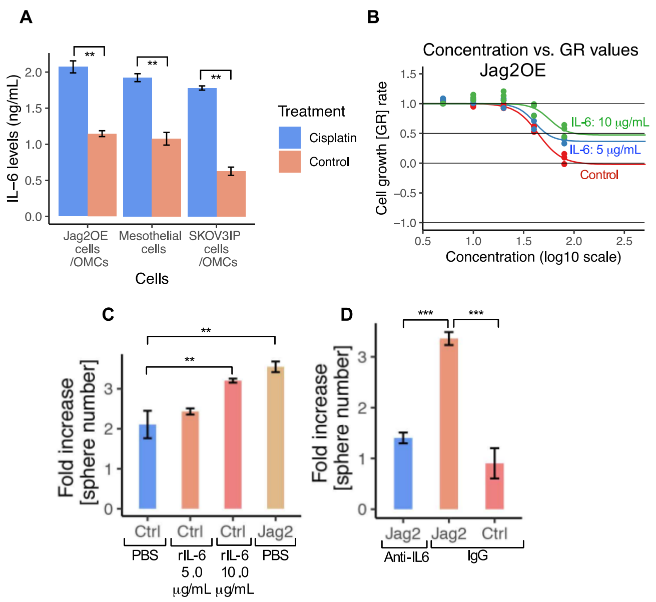

Supplementary Figure S5 of Figure 8

A Assessment of IL-6 levels in the conditioned media from the indicated tumor cells cocultured with mesothelial cells after treatment with cisplatin (10 μM) by ELISA. ***P* < 0.01.

B Cell viability of the indicated tumor cells co-cultured with mesothelial cells after treatment with 5 or 10 μg/ml of rIL- 6 and cisplatin.

C Quantification of the number of sphere growth of the indicated tumor cells co-cultured with mesothelial cells and treated with either PBS, 5.0 μg/mL, or 10.0 μg/mL of rIL6 antibody. ***P* < 0.01.

D Quantification of the number sphere growth of indicated tumor cells co-cultured with mesothelial cells and treated with either IgG or 5.0 μg/mL of anti-IL-6 antibody. ****P* < 0.001. Data in the figure represent average and +/-SEM; *P*-values were determined using the Student's *t*-test unless otherwise indicated. All experiments were run in triplicates.

Supplementary Figure S6 of Figure 10: Inhibiting the Notch signaling pathway in ovarian cancer cells with MRK-003 reverses Jagged-2 driven omental metastasis

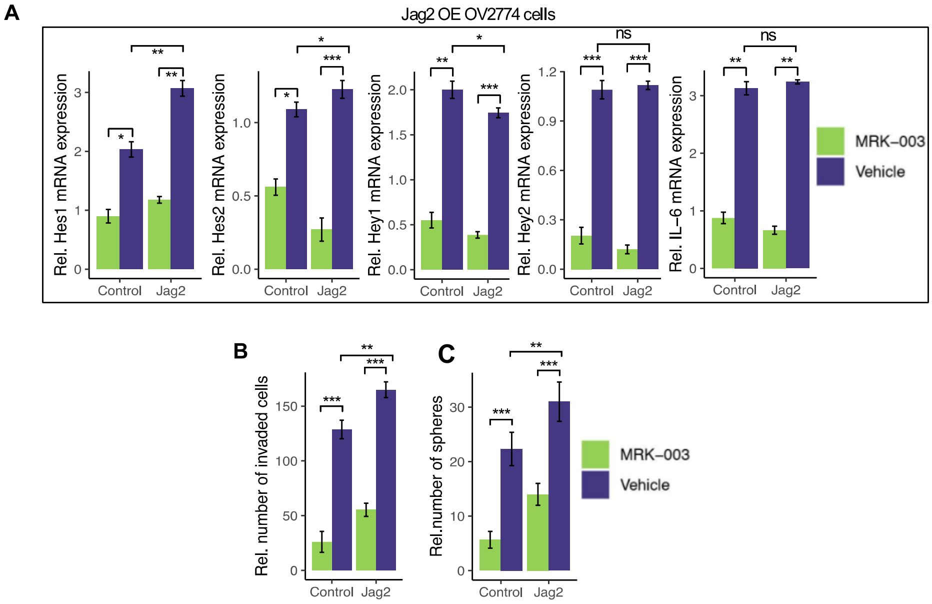

Supplementary Figure S6 of Figure 10

A qRT-PCR expression analysis of the Notch target genes and IL-6 from scraped-off cells from the omentum. mean +/- SEM, n = 3 for each experiment. *P < 0.05, **P < 0.01, ***P < 0.001, ns = not significant.

B-C Quantification of invasion and sphere formation capacity of Jag2OE cells in each experimental treatment group. **P < 0.01, ***P < 0.001.

Data in the figure represent average and +/-SEM; *P*-values were determined using the Student's *t*-test unless otherwise indicated. All experiments were run in triplicates.

Supplementary Table S1: List of primers

**Supplementary Table S1: List of primers**		
Genes	Forward primers – 5'-3'	Reverse primers – 5'-3'
CCND1	CTGATTGGACAGGCATGGGT	GTGCCTGGAAGTCAACGGTA
CCND2	GATTGAACCATTTGGGATGG	ATGGTGGTGTCTGCAATGAA
CCND3	AGGCTGATGGGACAGAATTG	ACCCCAATCCAAATGCAATA
CDK1	CCATGGGGATTCAGAAATTG	CCATTTTGCCAGAAATTCGT
CDK2	GCCCTAATCTCACCCTCTCC	AAGGGTGGTGGAGGCTAACT
CHK1	CAGGGGTGGTTTATCTGCAT	TTCCACAGGACCAAACATCA
CHK2	ATCCAAAGGCACGTTTTACG	ACAACACAGCAGCACACACA
RB1	TTGAGCACACGGTCGCTGTTAC	ATAGCCTCCTCCACGAACCT
P16/CDKN2A	TGCCTTTTCACTGTGTTGGA	GCCATTTGCTAGCAGTGTGA
P27/CDKN1B	ATAAGGAAGCGACCTGCAACCG	TTCTTGGGCGTCTGCTCCACAG
Jagged2	GTCAAGGTGGAGACGGTTGT	TGGTAGAGCACGTCCTTGTG
Notch1	CAACATCCAGGACAACATGG	GGACTTGCCCAGGTCATCTA
Notch2	AAGCAGAGTCCCAGTGCCTA	CAGGGGGCACTGACAGTAAT
Notch3	TGCTTCCTCTTTTGGCAGAT	TTCTTCTGCCCTGTTTGCTT
Notch4	CTAGGGGCTCTTCTCGTCCT	CAACTTCTGCCTTTGGCTTC
Hes1	CTCTCTTCCCTCCGGACTCT	AGGCGCAATCCAATATGAAC
Hes2	TAGCCTAAGGGCTCTGGTCA	GAAATTGATGCTCGGGAAAA
Hey1	CGAGGTGGAGAAGGAGAGTG	TCCTTGCTCCATTACCTGCT
Hey2	GAACAATTACTCGGGGCAAA	TCAAAAGCAGTTGGCACAAG
DLL1	TGTGCCTCAAGCACTACCAG	TTCTGTTGCGAGGTCATCAG
Jag1	GACTCATCAGCCGTGTCTCA	TGGGGAACACTCACACTCAA
Dll3	GAGACACCCAGGTCCTTTGA	CAGTGGCAGATGTAGGCAGA
Dll4	ACCTTTGGGTGTCTGTCTGG	ACTTTTGGAAACACGGATGC
FN1	ACCAACCTACGGATGACTCG	GCTCATCATCTGGCCATTTT
TGFB1	GGGACTATCCACCTGCAAGA	CCTCCTTGGCGTAGTAGTCG
IL-6	TACCCCCAGGAGAAGATTCC	TTTTCTGCCAGTGCCTCTTT
CTGF/CCN2	GGAAAAGATTCCCACCCAAT	TGCTCCTAAAGCCACACCTT
GAPDH	CGACCACTTTGTCAAGCTCA	AGGGGTCTACATGGCAACTG
SOX2	ACACCAATCCCATCCACACT	GCAAACTTCCTGCAAAGCTC
OCT3/4	GTACTCCTCGGTCCCTTTCC	CAAAAACCCTGGCACAAACT
Nanog	TTCCTTCCTCCATGGATCTG	TCTGCTGGAGGCTGAGGTAT
ALDH1A1	TGTTAGCTGATGCCGACTTG	TTCTTAGCCCGCTCAACACT

Table S2: Cell cycle analysis of control, co-culture, and CM-Meso cells

Table S2: Cell cycle analysis of control, co-culture, and CM-Meso cells

Cell lines	Culture conditions	G1 (%)	S (%)	G2/M (%)
SKOV3-ip	Control	40.5	36.4	23.1
	Co-culture	66.0	16.7	17.3
	CM-Meso	53.9	27.4	16.4
OVCAR3	Control	37.4	29.8	23.3
	Co-culture	43.3	20.5	21.1
	CM-Meso	51.2	26.3	22.4

CM-Meso: Conditioned medium from mesothelial cell cultures

Table S3: Cell cycle analysis of control, and Jagged2-overexpression (Jag2OE) OV2774 ovarian tumor cells under the treatment of DMSO and MRK-003

Table S3: Cell cycle analysis of control, and Jagged2-overexpression (Jag2OE) OV2774 ovarian tumor cells under the treatment of DMSO and MRK-003

Cell lines	Drug treatment	G1 (%)	S (%)	G_2/M (%)
Control	DMSO	43.2	31.7	25.1
Jag2OE	DMSO	42.4	32.6	25.1
Control	MRK-003	47.3	34.1	18.6
Jag2OE	MRK-003	45.8	32.7	21.5
