# Supplementary figures and images for: Ovarian tumor cell-derived JAGGED2 promotes omental metastasis through stimulating the Notch signaling pathway in the mesothelial cells
Source: Cell Death Dis. 2024 Apr 4;15(4):247. doi: 10.1038/s41419-024-06512-0 (PMC10995149; doi:10.1038/s41419-024-06512-0)

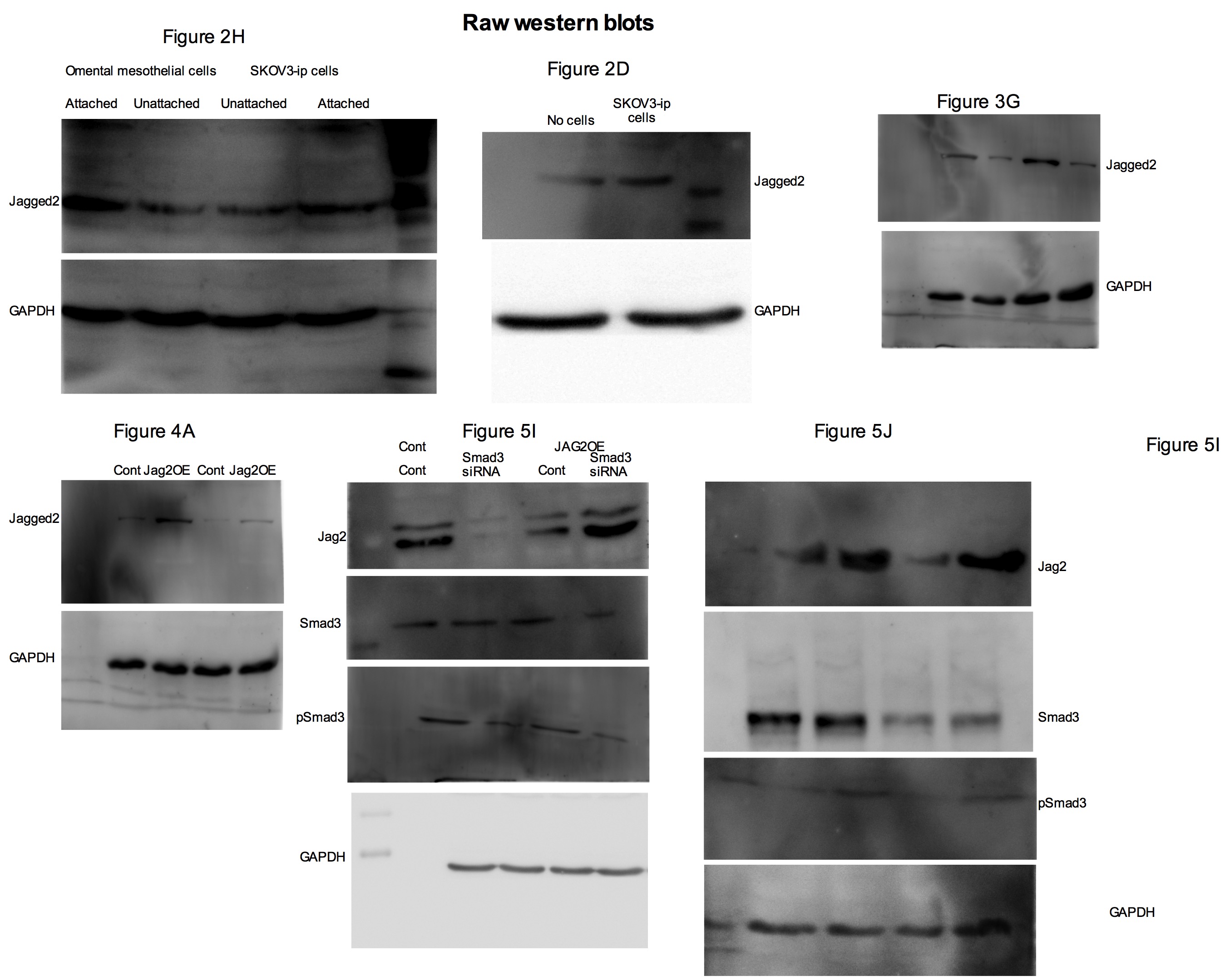

Supplement: Supplementary file 3 — Raw western blots [file 41419_2024_6512_MOESM3_ESM.jpg]
